# Transferability of genomic prediction models across market segments in potato and the effect of selection

**DOI:** 10.1007/s00122-025-05004-9

**Published:** 2025-08-20

**Authors:** Kathrin Thelen, Po-Ya Wu, Nadia Baig, Vanessa Prigge, Julien Bruckmüller, Katja Muders, Bernd Truberg, Stefanie Hartje, Juliane Renner, Delphine Van Inghelandt, Benjamin Stich

**Affiliations:** 1 Julius Kühn-Institute (JKI) – Federal Research Centre for Cultivated Plants, Institute for Breeding Research on Agricultural Crops, 18190 Sanitz, Germany; 2 Professorship for Utilization of Plant Genetic Resources for Breeding Purposes, Faculty of Agricultural- and Environmental Sciences, University of Rostock, 18051 Rostock, Germany; 3 Institute for Quantitative Genetics and Genomics of Plants, Heinrich Heine University, 40225 Düsseldorf, Germany; 4 SaKa Pflanzenzucht GmbH & Co. KG, 24340 Windeby, Germany; 5 Nordring-Kartoffelzucht- und Vermehrungs- GmbH, 18190 Sanitz, Germany; 6 EUROPLANT Innovation GmbH & Co. KG, 21337 Lüneburg, Germany; 7 Leibniz Institute of Plant Genetics and Crop Plant Research (IPK), 06466 Seeland, Germany

## Abstract

Genomic prediction (GP) can help increase the efficiency of breeding programs, as genotypes can be selected based on their predicted performance. However, to the best of our knowledge, this procedure is not yet routine in commercial breeding programs in tetraploid organisms like potato (*Solanum tuberosum* L.). The objectives of this study were to

(i) Estimate the prediction accuracy for 26 different potato traits in a panel of about 1000 genotypes based on 202,008 single nucleotide polymorphisms,
(ii) Evaluate the influence of the size and constitution of the training set on the prediction accuracy, and
(iii) Investigate how the effect of selection in the training set influences the outcome of GP.

GP revealed high prediction accuracies using genomic best linear unbiased prediction. Our results indicated that a training set of 280–480 clones and 10,000 markers was sufficient. Prediction within a specific market segment led to a higher prediction accuracy compared to adding clones from other market segments to the training set or to predict between different market segments. Lastly, we found a higher prediction accuracy when in a training set of selected clones, i.e., a training set that consists of clones with high trait values, 20% of the clones were replaced by clones that were sampled from the clones that showed the lowest 10% trait values. This observation shows that clones from advanced breeding stages can be used as training set, if some clones specifically from the other side of the distribution range are added to the training set.

**Supplementary Information:**

The online version contains supplementary material available at 10.1007/s00122-025-05004-9.

## Introduction

Potato (*Solanum tuberosum* L.) is one of the most important food crops worldwide (Devaux et al. 2020; FAOSTAT database 2024). Its high nutrient content, together with its high amount of carbohydrates (Reddy et al. 2018), makes it an essential component of the diets of one billion people worldwide (Devaux et al. 2020). Potato can be grown in many different environments under temperate, subtropical, and tropical conditions (Devaux et al. 2020; Bradshaw 2017). Besides, it is a versatile short-duration crop whose demand is steadily increasing (Reddy et al. 2018). Potato has various market segments, such as fresh consumption, processing to crisps or French fries, or starch production. Potato breeding programs aim for the development of specific cultivars suited for those different market segments (Bradshaw 2017). The breeding progress made for relevant traits in the past is limited by the autotetraploidy of potato, as well as its high heterozygosity and inbreeding depression (Naeem et al. 2021). Furthermore, more than 40 different traits have to be evaluated during the selection process (Slater et al. 2013). Some of these traits are evaluated relatively late in the breeding process, especially because the number of available tubers is low in the early stages of the selection process and the evaluation of some traits includes destructive measurements (Gebhardt 2013), and/or requires multi-environment testing (Jansky 2009).

Genetic gain can be improved by using molecular genetic markers like single nucleotide polymorphisms (SNPs) for selection (Crossa et al. 2017). This is especially true as the genotyping costs were notably reduced over the past years and will likely continue to drop (Slater et al. 2013; Crossa et al. 2017). One promising approach of using molecular markers for selection of quantitative traits is genomic prediction (GP). GP, which was originally proposed by Meuwissen et al. (2001), uses genome-wide marker effects estimated in a phenotyped and genotyped training set (TS) to estimate so-called genomic estimated breeding values (GEBVs) of a validation set (VS) that is only genotyped. This reduces the time as well as the costs of one breeding cycle (Slater et al. 2016), as selection can be performed in earlier stages of the breeding program based on the predicted performance of the clones and thereby lowers the phenotyping costs (Sverrisdóttir et al. 2018). Thus, the implementation of GP can increase the gain of selection, which has also been illustrated for potato based on computer simulations (Wu et al. 2023). GP was originally designed for animal breeding (Meuwissen et al. 2001), but its implication in plant breeding has been studied intensively during the last decades (for review see Crossa et al., 2017; Alemu et al., 2024). However, studies focusing on the implementation of GP in tetraploid organisms like alfalfa (Li et al. 2015), ryegrass (Guo et al. 2018), blueberry (De Bem Oliveira et al. 2020), and potato (e.g., Slater et al. 2016; Sverrisdóttir et al. 2018; Habyarimana et al. 2017; Selga et al. 2022, ) are less abundant. Particularly empirical analyses of prediction accuracy in large sets of tetraploid potato clones for a wide range of commercially relevant traits in breeding programs of potato are largely missing.

One important step in the implementation of GP is the establishment of the TS. Several studies focusing on the optimization of the TS, mainly in diploids (e.g., Rio et al. 2021; Akdemir et al. 2015; Isidro et al. 2015, ), and a few in tetraploids (e.g., Sverrisdóttir et al. 2018; Wilson et al. 2021a, ) have been published. These studies aimed at optimizing the composition of the training set based on the known genomic relationship among the individual entries. Wilson et al. (2021a) found that including genetically more similar clones in TS and VS led to better predictions in tetraploids. Brauner et al. (2020) found that using unrelated genotypes decreases the prediction accuracy in maize, while Norman et al. (2018) found that the accuracy can be improved by increasing the diversity of the TS in wheat. Sverrisdóttir et al. (2018) found that adding individuals from more distant populations to the training set will not increase the PA for the traits chipping quality and dry matter content.

In potato breeding programs, clones from different market segments are available in each breeding stage. However, to the best of our knowledge, it is not clear if and how to combine these clones from different market segments in the context of TS establishment. In addition, only little is known about the effect of a preselection for target traits in potato, or how promising it is to include in the TS genotypes that would not have been advanced in a breeding program.

The objectives of this study were to

(i) Estimate the prediction accuracy for 26 different potato traits in a panel of about 1,000 genotypes based on 202,008 SNPs,
(ii) Evaluate the influence of the size and constitution of the training set on the prediction accuracy, and
(iii) Investigate how selection in the TS can influence the outcome of GP.

## Materials and methods

### Genetic material

The plant material of this study was a subset of the potato breeding populations from the breeding companies SaKa (SaKa Pflanzenzucht GmbH & Co. KG), Norika (Nordring-Kartoffelzucht- und Vermehrungs- GmbH), and Europlant (EUROPLANT Innovation GmbH & Co. KG). Overall, 1066 tetraploid potato clones corresponding to the A clone level (*cf.* Stich & Van Inghelandt 2018) were used in our study. These clones belong to 171 full-sib families, which were designated in the following as segregating populations. The number of clones within one population varied from one to 38 clones (Fig. S1).

The above-mentioned 1066 clones were evaluated in the years 2019, 2020, and 2021 in field experiments in different locations in Germany. Each breeding company evaluated their proprietary clones (i.e., entries) together with eight common variety checks. This experimental design was chosen due to intellectual property and phytosanitary reasons. The evaluated entries represent four different market segments, which correspond to the main usage groups of potatoes in Europe: crisp production (CR), French fries production (FF), starch production (ST), and table potato (TA). The number of clones within one market segment varied among the segments, with ST being the smallest (150 clones), followed by TA (263 clones) and FF (266 clones), while CR was the largest group with 379 clones. The assignment of clones to the market segments was conducted based on the parental market segment as cross purpose as defined by the responsible breeder.

In order to examine the effect of preselection on the prediction accuracy of GP, 330 out of the overall 1066 genotypes corresponded to clones that would have normally been discarded in commercial breeding programs in the single hills stage based on breeder’s selection on different trait combinations for the four different market segments (Table S1). This group of clones, in the following designated as clones with discard status, comprised clones from biparental families that would have normally been discarded completely (68 clones, discard status 2), as well as clones from biparental families, where other clones from the same family were retained as A clones (262 clones, discard status 1).

Clones were evaluated at one location in 2019, which was Kaltenberg for Europlant, Groß Lüsewitz for Norika, and Windeby for SaKa. In 2020 and 2021, an additional location was added for each breeding company, which was Böhlendorf for Europlant, Mehringen for Norika, and Gransebieth for SaKa. This resulted in five different year-location combinations for each of the three breeding companies, which were designated in the following as environments (Table S2).

In general, more clones were evaluated in 2019 than in 2020 and 2021, which was a result of virus infections in multiplication plots that lead to the unavailability of clones in the 2020 and 2021 experiments due to phytosanitary requirements. Within the environments, the clones were organized in a block system following an augmented experimental study design. SaKa organized their clones in eight different blocks, whereas Europlant and Norika each organized their clones in up to four different blocks. The blocks were further organized into rows and columns. The eight checks were replicated eight times in each environment, at least one time in each block, while the entries were cultivated only once within each environment of their donor breeding company. Thus, each entry was replicated five times in total across all environments. The number of plants per plot ranged from nine to 20, depending on the respective environment (Table S2). In 2021, the experiment from the breeding company SaKa was further organized in two different trials, which split the clones according to their foliage maturity group. One trial contained clones from the extra early to early maturity group, and the other trial contained clones from the middle early to middle late maturity group. The two trials were adjacent to one another in the field and each trial contained the eight check clones in each block.

Data were recorded on an individual plot basis for 26 different traits (Table 1). Trait values were either assessed as a rating from 1 to 9 or given in the form of a percentage value. The traits were assessed using methods that were commonly used by the three breeding companies (Table 1) and are standardized techniques, so that the breeding companies minimized the level of subjectivity within and between the environments. In addition, the ratings were either performed by one person per environment or one person per block. Furthermore, the total tuber yield per plot ($\textrm{YLD}_{\textrm{raw}}$) was measured in kilograms and corrected to a tuber yield per plot of 16 plants ($\textrm{YLD}$) based on the following model, which is employed in the commercial breeding programs (Truberg, personal communication):

1 $$\begin{aligned} \textrm{YLD} = \frac{\textrm{YLD}_{\textrm{raw}}}{\textrm{PN} - \textrm{MP}_{ > 20\% }} *16 \,, \end{aligned}$$

where PN is the number of plants planted for the corresponding plot and MP is the amount of missing plants in the plot. Here, MP was only subtracted from PN if MP exceeded 20% of PN, as for a lower extent of MP a full compensation of the remaining plants is expected (Truberg, personal communication). The plot size was set to 16 plants, as this was across all environments the most frequently used plot size (Table S2).

Due to limitations of the number of available tubers in 2019, the traits BRU, CR4, CR8, DSC, FRI, TEX, TST, TUL, TUN, and TUS were only assessed for the second and third year (2020 and 2021), while the other traits were assessed in all three years. The traits CR4 and FRI were not evaluated for all clones, but only for those clones that belonged to the specific market segment, which was CR for CR4 and FF for FRI.

### Statistical analyses

In general, data preprocessing and phenotypic data analyses were performed as described in detail by Thelen et al. (2025). Briefly, the following analyses were carried out. In a first step, the data of the breeding company SaKa from 2021 were corrected for the trial effects of the two trials. This was done for each trait individually by first calculating the mean values of the eight checks for each trial. Then, the absolute value of the mean difference between the checks of both trials was subtracted from each observation of the trial with the higher mean value.

In the following analyses, the breeding companies were denoted as B1, B2, and B3 and the different locations for each breeding company were given by L1 and L2. Potential outliers were then identified by fitting model (2) to the complete data set. As only checks were replicated in each environment, the genotype–environment interaction effect could only be estimated for the checks:

2 $$\begin{aligned} y_{ijklm} = \mu + g_{i} + e_{j} + C_{i}(ge)_{ij} + b_{kj} + r_{lkj} + h_{mkj} +\epsilon _{ijklm} \,, \end{aligned}$$

where $y_{ijklm}$ is the phenotypic observation of the *i*th potato clone in the *m*th column and the *l*th row of the *k*th block in the *j*th environment, $\mu$ an intercept term, $g_{i}$ the effect of the *i*th clone, $e_{j}$ the effect of the *j*th environment, $C_{i}$ a dummy variable filtering for checks with $C_{i} = 1$ for checks and $C_{i} = 0$ for entries, $(ge)_{ij}$ the interaction effect of the *i*th clone and the *j*th environment, $b_{kj}$ the effect of the *k*th block of the *j*th environment, $r_{lkj}$ the effect of the *l*th row of the *k*th block of the *j*th environment, $h_{mkj}$ the effect of the *m*th column of the *k*th block of the *j*th environment, and $\epsilon _{ijklm}$ the residual error. Except for $g_{i}$, all effects were considered random. Based on this analysis for each trait, records with a standardized absolute residual value greater than 3.5 were considered as outliers and were removed from the data set.

In the next step, a correction for the check-based block effect was realized as described for the trial effect in case of a significant ($\alpha$ = 0.05) likelihood ratio test (LRT) in model (2). The corrected trait values were used for all further analyses.

Adjusted entry means (AEMs) for all clones (checks and entries) were calculated across all environments. When doing so, the residual error variance was assumed to be heterogeneous across the different environments (Thelen et al. 2025).

The genotypic variance of the entries was calculated by model (3), where the clone effect was split up between checks and entries:

3 $$\begin{aligned} y_{ijklm} = \mu + C_{i}g_{i} + D_{i}g_{i} + e_{j} + C_{i}(ge)_{ij} + b_{kj} + r_{lkj} + h_{mkj} +\epsilon _{ijklm} \,, \end{aligned}$$

where $D_{i}$ is an indicator variable filtering for entries with $D_{i}$=0 for checks and $D_{i}$= 1 for entries. Thereby, the effect of the checks was regarded as fixed and the effect of the entries was regarded as random. Again, the residual error variance was assumed to be heterogeneous across the different environments.

Heritability on an entry mean basis was calculated for each trait according to the following formula based on the entries (Piepho & Möhring 2007):

4 $$\begin{aligned} h^{2} = \frac{\sigma _{g}^{2}}{\sigma _{g}^{2} + \frac{\hat{\nu }}{2}} \,, \end{aligned}$$

where $\sigma _{g}^{2}$ is the genotypic variance of the entries estimated by model (3) and $\hat{\nu }$ the mean variance of a difference of two adjusted treatment means of the entries as they were estimated by model (2).

### Genomic prediction

Out of the 1066 different clones included in the field experiment described above, 988 have been genotyped using an SNP array (Baig et al., in preparation). The overall 224,009 high-quality SNP markers were filtered according to the following criteria:

(i) Loci had < 20% missing data, and
(ii) Loci had a minor allele frequency $\ge$ 0.05.

A total of 202,008 SNP markers remained after quality preprocessing of the complete data set. The 2.9% missing SNP data points were mean imputed.

GP was performed using the univariate genomic best linear unbiased prediction (GBLUP) model, which can be described as

5 $$\begin{aligned} \textbf{y} = \mathbf {1_{n}}\mu + \mathbf {Z_{a}a} + \mathbf {\epsilon } \,, \end{aligned}$$

where $\textbf{y}$ is a vector of the AEMs calculated from model (2) for each trait, $\mathbf {1_{n}}$ a vector of ones, $\mu$ the overall mean, $\mathbf {Z_{a}}$ the design matrix mapping AEMs to clones, $\textbf{a}$ the vector of genetic values contributed by additive effects that are assumed to be normally distributed with $N(0,\mathbf {G_{A}}\sigma ^{2}_{a})$, in which $\mathbf {G_{A}}$ was the genomic relationship matrix calculated according to VanRaden (2008) and extended following Ashraf et al. (2016) using an additive autotetraploid model, and $\mathbf {\epsilon }$ the vector of residual, assumed to be normally distributed $N(0,\mathrm {\textbf{I}} \sigma ^2_\epsilon )$, where $\mathrm {\textbf{I}}$ is an identity matrix and $\sigma ^2_\epsilon$ the residual variance.

In the next step, the model was extended by including dominance effects:

6 $$\begin{aligned} \textbf{y} =\mathbf {1_{n}}\mu + \mathbf {Z_{a}a}+ \mathbf {Z_{d}d} + \epsilon \,, \end{aligned}$$

where $\mathbf {Z_{d}}$ is the design matrix mapping AEMs to clones, $\textbf{d}$ the vector of genetic values contributed by dominance effects, distributed as $N(0,\mathbf {G_{D}}\sigma ^{2}_{d})$, where $\sigma ^{2}_{d}$ is the dominance variance and $\mathbf {G_{D}}$ the dominance relationship matrix. In this study, the dominance effects, which are the deviation of the observed genetic value from the expected based on the pure additive model, were set differently for the three heterozygous genotypes (Aaaa, AAaa, and AAAa) and were designated by $\textbf{d1}$, $\textbf{d2}$, and $\textbf{d3}$, respectively. This procedure resulted in the three separate dominance effect matrices $\mathbf {X_{d1}}$, $\mathbf {X_{d2}}$, and $\mathbf {X_{d3}}$ with a dimension $n \times m$, in which *n* was the number of clones and *m* the number of SNP markers. For $\mathbf {X_{d1}}$, clones carrying Aaaa were coded as 1 and the others 0; for $\mathbf {X_{d2}}$, clones carrying AAaa were coded as 1 and the others 0, and for $\mathbf {X_{d3}}$, clones carrying AAAa were coded as 1 and the others 0 (Gallais, 2003; Table 2 in Wu et al., 2025). Then, $\mathbf {X_{d}}$ was defined as a combinatorial matrix of the three dominance effects matrices, expressed by $\mathbf {X_{d}} = [\mathbf {X_{d1}},\mathbf {X_{d2}}, \mathbf {X_{d3}}]$, and was used to calculate $\mathbf {G_{D}} = \frac{\mathbf {X_{d}X_{d}}^T}{3\,m}$.

For the implementation of the dominance effect, we chose a median imputation in order to have a unique assignment of genotypes to dominance effects.

The GBLUP models were implemented using the R packages AGHmatrix (Amadeu et al. 2023) and sommer (Covarrubias-Pazaran 2016).

The effects of the model were estimated within a TS and then used to estimate the GEBVs of the genotypes of the VS. If not mentioned differently, a fivefold cross-validation (CV) approach was used in our study. For this procedure, the data were randomly split into five parts, and one part was used as the VS, while the other four parts were used as TS. Each of the five parts was independently used as VS once.

Model performance for each individual trait was measured by the prediction accuracy (PA), which was the Pearson correlation coefficient between the AEMs from equation (2) and the GEBVs of the VS, divided by h (the square root of the heritability), to account for differences in the predictions which are due to differences in the heritability. Different modifications of the TS and VS were analyzed as described below.

**Modifications of TS and VS**

*Analysis of the complete data set:* In a first step, GP was applied to the complete data set with all available genotypes and markers. The CV process was replicated 50 times. This analysis was also used to assess the influence of dominance effects as described above in models (5) and (6). If the model resulted in a singularity, the calculation was canceled. In all further scenarios, only model (5) was used.

*Effect of the size of the training set:* The influence of the size of the TS was examined using different random subsets of the complete data set. The subset sizes of the data set ranged from 50 to 950 in steps of 50 and, thus, the resulting TS sizes *N* ranged from 40 to 760 in steps of 40. The CV process was replicated 50 times, and for each replication, a new subset of the respective size was sampled from the complete data set.

*Effect of the number of markers:* The influence of the number of SNPs on the PA was explored by using a stratified sampling approach. The genome was split into equally sized windows, and from each window one marker was randomly chosen. In different sub-scenarios, the window size was doubled and, thus, increased from 2 (set with 101,004 markers) to 1024 (197 markers). For each subset of markers, all available genotypes were used, and the fivefold CV was performed with 50 replications for each size scenario. For each replication, new SNPs were sampled.

*Effect of the variability in the number of clones per segregating population: * In this study, the size of the different segregating populations differed considerably. Thus, to evaluate the effect of this unbalancedness, we compared the PA of the original unbalanced set, to a set with balanced population sizes. For the balanced set, four clones were taken randomly from each population to build the TS. Populations with less clones were (i) fully included or (ii) excluded. The respective TS sizes were defined by the number of populations with/without more than four clones and were for i) 457 clones and for ii) 268 clones. These sizes were then used to build an unbalanced TS with the contribution of the individual populations as in the complete set, by using a stratified sampling approach across populations. Thereby, for each TS, the clones of each population had the same proportion as in the complete data set, but TS sizes were comparable to scenario i) and ii). Again, clones from smaller populations were iii) included fully (TS size of 474) or iv) excluded (TS size of 257). For the random scenario, the v) 450 and vi) 270 clones were sampled randomly from the complete set of clones. The VS comprised 120 random clones not present in the TS and were the same across the three scenarios of similar sizes for the respective replication. Each procedure was repeated 50 times.

**Fig. 1:**
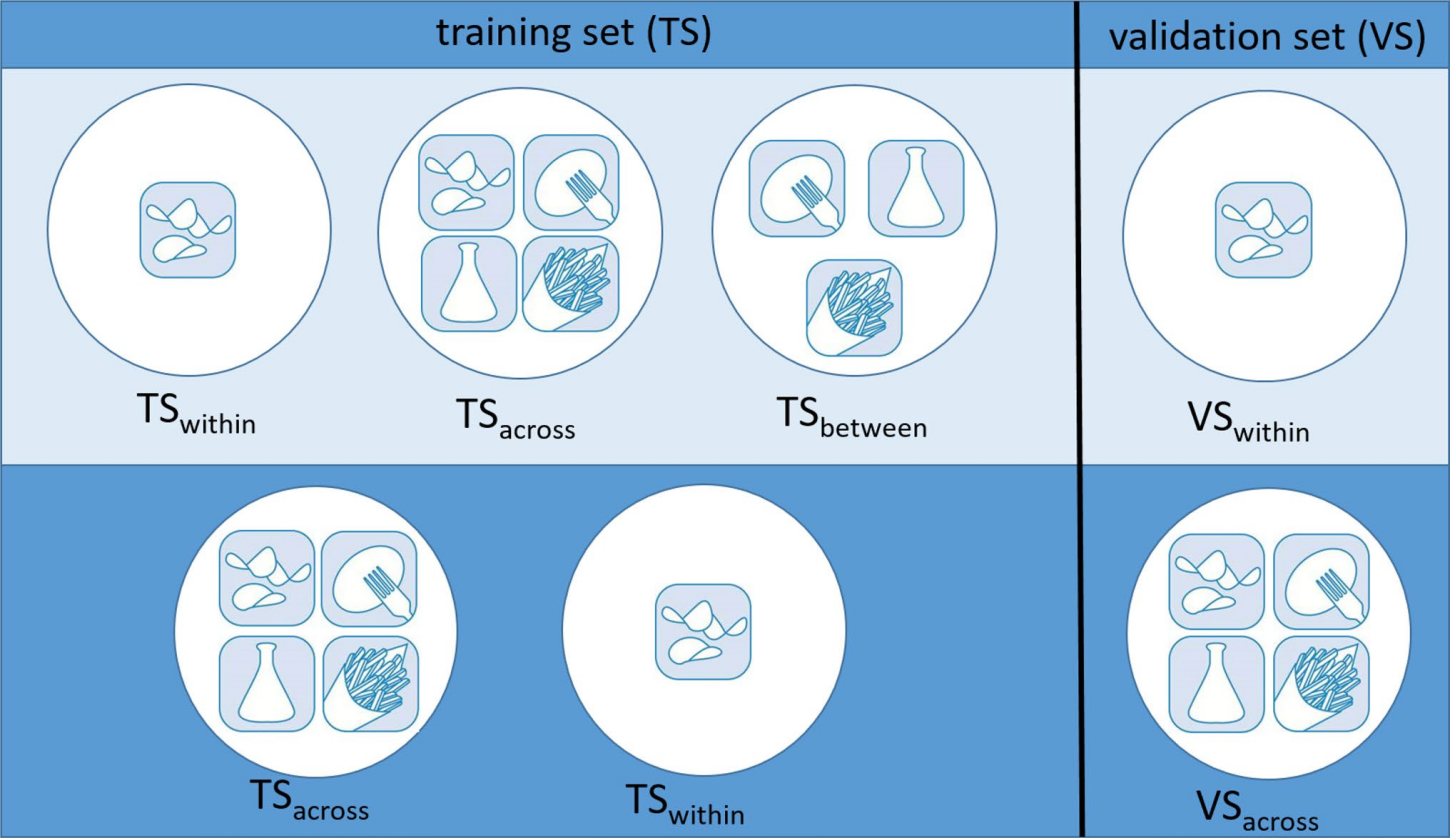
Schematic diagram of the different prediction scenarios evaluated in our study to examine the transferability of prediction models among genetic groups, i.e., market segments, which were represented here by four different icons. The two main perspectives are depicted in the two rows and differ by the analyzed validation set (VS). In the first case of a $\textrm{VS}_{\textrm{within}}$, the clones of the VS are from only one market segment, exemplified by the crisps potato. In the second perspective of a $VS_{\textrm{across}}$, the clones in the VS come from all four market segments (French fry potato, crisps potato, starch potato, and table potato). Both perspectives can be trained with different training sets (TS). The first perspective was analyzed in the case of $\textrm{TS}_{\textrm{within}}$ when clones in the TS are from the same market segment as clones in the VS. In case of $\textrm{TS}_{\textrm{across}}$ clones in the TS are from all market segments. In case of $\textrm{TS}_{\textrm{between}}$, clones in the TS are from all market segments except the one in the VS. For the second perspective, clones in the TS are in the case of a $\textrm{TS}_{\textrm{across}}$ again from all market segments like in the VS, and in the case of a $\textrm{TS}_{\textrm{within}}$ from only one of the market segments. In the $\textrm{TS}_{\textrm{within}}$ scenarios, the market segments crisps potato, French fry potato, and table potato were considered separately

*Transferability of GP models across market segments:* Within a breeding program that develops genetic material for different market segments, two different perspectives for the transferability of GP models across market segments exist (Fig. 1). In the first perspective, breeders aim for the best PA of clones of one specific market segment. In this scenario, designated in the following as $\textrm{VS}_{\textrm{within}}$, the clones in the VS were explicitly taken from one market segment. Here, several sub-scenarios were examined. In the sub-scenario $\textrm{TS}_{\textrm{within}}$, the clones in the TS belonged to the same market segment as the clones in the VS. However, we also examined in the sub-scenario $\textrm{TS}_{\textrm{across}}$ the influence of other clones in the TS. In this case, the clones in the TS were randomly chosen across all market segments but at the same time, the VS was kept constant. In the sub-scenario $\textrm{TS}_{\textrm{between}}$, the TS was a subset of clones from three market segments to equal shares, while the VS comprised the clones of the fourth market segment. The TS size was set to 200 clones; thus, the before-described analyses were only performed for VS comprising clones from TA, FF, or CR. The VS comprised 45 clones and the analyses were run with 150 replications. The traits CR4 and FRI were excluded from this analysis, as they were only evaluated in their own market segments.

In addition to a prediction where only clones from one market segment are part of the VS, in the context of a breeding program it is also relevant to realize GP with a VS consisting of different market segments. In this second perspective, the clones of the VS were sampled across different market segments instead of within one market segment. This scenario was called $\textrm{VS}_{\textrm{across}}$, which means that the clones in the VS were randomly sampled across all market segments. The performance of the clones from this VS was predicted from TS of clones of one market segment but also from TS that were assembled across market segments.

In another scenario named $\textrm{TS}_{\mathrm{between-pairwise}}$, the before-described $\textrm{VS}_{\textrm{within}}$-$\textrm{TS}_{\textrm{between}}$ market segment analysis was also performed in a one-to-one comparison for various pairs of market segments: Clones from a single market segment were used as TS to predict the clones of another market segment. The number of replications for this variant was set to 15. Here again, the TS size was set to 200 clones.

*Transferability of GP models across breeding companies:* The PA was also compared for predictions of the different breeding companies. Like for the market segments, two scenarios were examined, where the first scenario was the $\textrm{VS}_{\textrm{within}}$ breeding company scenario. Here, the VS comprised only clones of one breeding company, leading again to the three sub-scenarios $\textrm{TS}_{\textrm{within}}$ (TS and VS from the same breeding company), $\textrm{TS}_{\textrm{across}}$ (TS as a sample from all breeding companies), and $\textrm{TS}_{\textrm{between}}$ (TS from the other breeding companies, excluding clones from the breeding company in the VS). The second scenario was the $\textrm{VS}_{\textrm{across}}$ breeding company analysis. In this case, the VS was made up of clones from all breeding companies and the TS comprised clones of only one breeding company or clones from all breeding companies. All analyses were performed with identical sizes and proportions of the TS as for the market segment analyses.

*Effect of truncation selection:* We analyzed the effect of trait-specific selection on the PA. To exemplify this selection, we selected for each sampling round clones of the TS belonging to the x% clones with the highest trait values. This does not necessarily correspond to the fraction selected by breeding companies but should not influence our conclusion. x ranged from 30 to 70 in steps of 10. Additionally, y% of each TS was exchanged by clones that did not belong to the clones with the x% highest trait values, where y ranged from 0 to 30 in steps of 10. For each combination of x and y values, 50 replications were performed. All clones that were not part of the TS were part of the VS. For this study, we used the four example traits STA, FLE, YLD, and EMR, which cover the whole range of high, medium, and low PA.

To furthermore evaluate the influence of the type of exchanged clones, we compared for the scenario y=20% different sampling methods:

(i) Random sampling among all clones that did not belong to the set of clones with the x% highest trait values (as described above),
(ii) Stratified sampling among all clones that did not belong to the set of clones with the x% highest trait values. For stratification, the entire phenotypic range of a trait was divided into ten equally sized windows. The clones were then randomly selected to complement the TS from those windows that did not comprise clones of the TS. Each window contributed the equal amount of clones to realize a uniform distribution of the previously not represented windows. And
(iii) Sampling the clones from the set of clones with the lowest 10% trait values.

Statistical differences between the sampling methods of the exchanged clones with respect to the PA were evaluated by using a t test with Bonferroni correction.

Breeders do not select their clones based on one trait alone, but on a combination of different traits. Thus, to evaluate this influence, we extracted the rank of each clone for each trait and then added up the ranks of the four above-mentioned traits STA, FLE, YLD, and EMR. This final rank value across the four traits was then used to define the top 50% and the bottom 10% of clones, which were used to build the TS and redo the above-described analyses. We examined the PA from a TS sampled from clones in the top 50% ranks and exchanged 0% or 20% in the TS with clones that belong to the bottom 10% ranks. Furthermore, we compared the PA of these analyses with the PA of the analyses with single trait values as described before that had the same values for x and y.

*Effect of selection on correlated traits:* In a last step, we investigated if a previous selection on correlated traits of the examined clones influenced the PA. This was done by building a TS from subsets of the clones, including different amounts of clones with discard status. Clones with discard status 1 would normally have been discarded in the single hills stage based on their phenotype, but other clones of their population were retained as A clones. To remove the influence of the number and sizes of different populations, a stratified sampling approach was used. Thus, in the first scenario, clones without discard status (i.e., the clones that were in fact advanced in the single hills stage in the commercial breeding program) were sampled according to their population structure so that each population contributed proportionally as many clones to the reduced TS as they contributed to the entire set of clones. For the further scenarios, for each sampling round, *Z* clones without discard status were exchanged from the TS by clones with discard status 1, while keeping the contribution from each population to the TS constant. *Z* ranged from 10% to 50% of the TS in steps of 10% where the TS size was 200 clones. Each scenario was replicated 150 times. The VS only consisted of clones without discard status for this particular analysis.

In order to check for the presence of market segment effects and the removal of these effects, an analysis including 0%, 20%, and 40% of clones with discard status 1 in the TS was also performed exclusively for clones from one market segment, namely CR. The market segment CR was used, as this was the biggest market segment with 235 clones with discard status 0, which led to a TS size of 188 clones in the fivefold CV. Here, the exchange was not performed within the families, but 0%, 20%, and 40% of clones without discard status were randomly replaced by clones with discard status 1. A total of 50 replications were used for each scenario.

**Diversity of TS and VS**

In order to evaluate to what extent the PA depends on the genetic distance between TS and VS, the genetic distance (Nei & Roychoudhury 1974) between each TS and VS was calculated for each prediction of the market segment analysis and the breeding company analysis using the R package StAMPP (Pembleton et al. 2013). Furthermore, to evaluate to which extent the clones in the TS or the VS vary genetically, the gene diversity for each TS and VS, quantified by the expected heterozygosity ($\mathrm {H_e}$), was calculated as:

7 $$\begin{aligned} \mathrm {H_e} = \frac{1}{m} \sum ^{m}_{j=1}\left( 1-\sum _{i=1}^{2} p_{i(j)}^4\right) \,, \end{aligned}$$

where *m* is the number of markers, and $p_{i(j)}$ the allele frequency of the *i*th allele at the *j*th marker (Gallais 2003; Wu et al. 2025). $\mathrm {H_e}$ was also calculated for the set of parental clones. To compare this estimate with the gene diversity of the non-parental clones while accounting for differences in the set sizes, we randomly sampled 150 times 121 clones, which corresponds to the number of parental clones, from the set of non-parental clones and averaged the observed $\mathrm {H_e}$.

Phenotypic diversity was measured in our study by calculating the variance of the AEMs of the clones in the TS and the VS. The diversity measures were averaged across all replications from the same analysis, and statistical significance was evaluated by a t test with Bonferroni correction. $Q_{ST}$ (Spitze 1993) is a statistic intended to measure the degree of genetic differentiation among populations with regard to a quantitative trait. It was calculated as the proportion of the genetic variance among the market segments to the complete genetic variance, which was the variance among the market segments plus the variance within the market segments. To check for the presence of structure in the data, a principal component analysis (PCA) was performed using the R package ade4 (Dray & Dufour 2007). Furthermore, the global fixation index ($F_{st}$) was calculated according to Nei (1986) where for $H_t$ the complete data set was considered as one large meta population, and for $H_s$ the average expected $H_e$ of the four market segments was calculated. Pairwise $F_{st}$ values between the market segments were calculated using the R package StAMPP (Pembleton et al. 2013).

If not mentioned differently, all analyses were performed using the software R, version 4.2.1 (R Core Team 2022).

## Results

Population structure based on SNP information was examined by a PCA, where the first two principal components explained 3.4% and 3.1%, respectively. Overall, four clusters were visible (Figs. S2 and S3). Two smaller clusters consisted exclusively of clones from one market segment, namely TA and ST. The clones from the market segment CR clustered together with the remaining clones of the market segment ST, while the clones from the market segment FF clustered together with clones from TA and CR (Figure S2). Furthermore, the two smaller clusters completely consisted of clones from breeding company 2, while no clear structure was observed in the other two clusters regarding the breeding company (Figure S3). The global $F_{st}$ was 0.01, and the pairwise $F_{st}$ values of the clones per market segment ranged between 0.01 and 0.02 (Table S3). The mean expected heterozygosity from 150 replications of 121 random tested clones was with 0.58 the same as that of the parental clones.

**Fig. 2:**
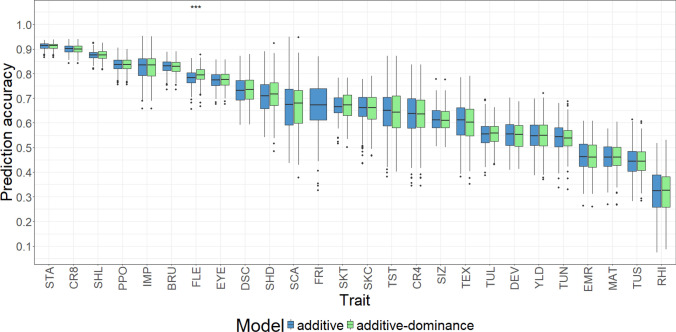
Boxplot of the prediction accuracy (PA) of the complete data set for the 26 evaluated traits using the additive (blue) and additive-dominance (green) models. For each trait, a fivefold cross-validation with 50 replications was used. Significant differences of the PA between the models of one trait are indicated by ***. For abbreviations of the traits, see Table 1

In the next step, GP was carried out using the complete data set using a univariate additive and an additive-dominance model. Except for FLE, no significant improvement using the additive-dominance model was observed compared to the additive model (Fig. 2). The highest PAs with values between 0.84 and 0.91 were found for the traits STA, CR8, SHL, and PPO. Overall, 22 out of 26 traits had a median PA above 0.5. Only EMR, MAT, TUS, and RHI had a median PA below 0.5. The median PA for tuber yield was 0.55. We observed that traits with a higher median PA also varied less around their median in the single CV runs compared to the traits with a lower median PA. A general high dispersion of the PA values was found for SCA, FRI, TST, CR4, TEX, and RHI.

*Effect of TS size and SNP number on PA:*

**Fig. 3:**
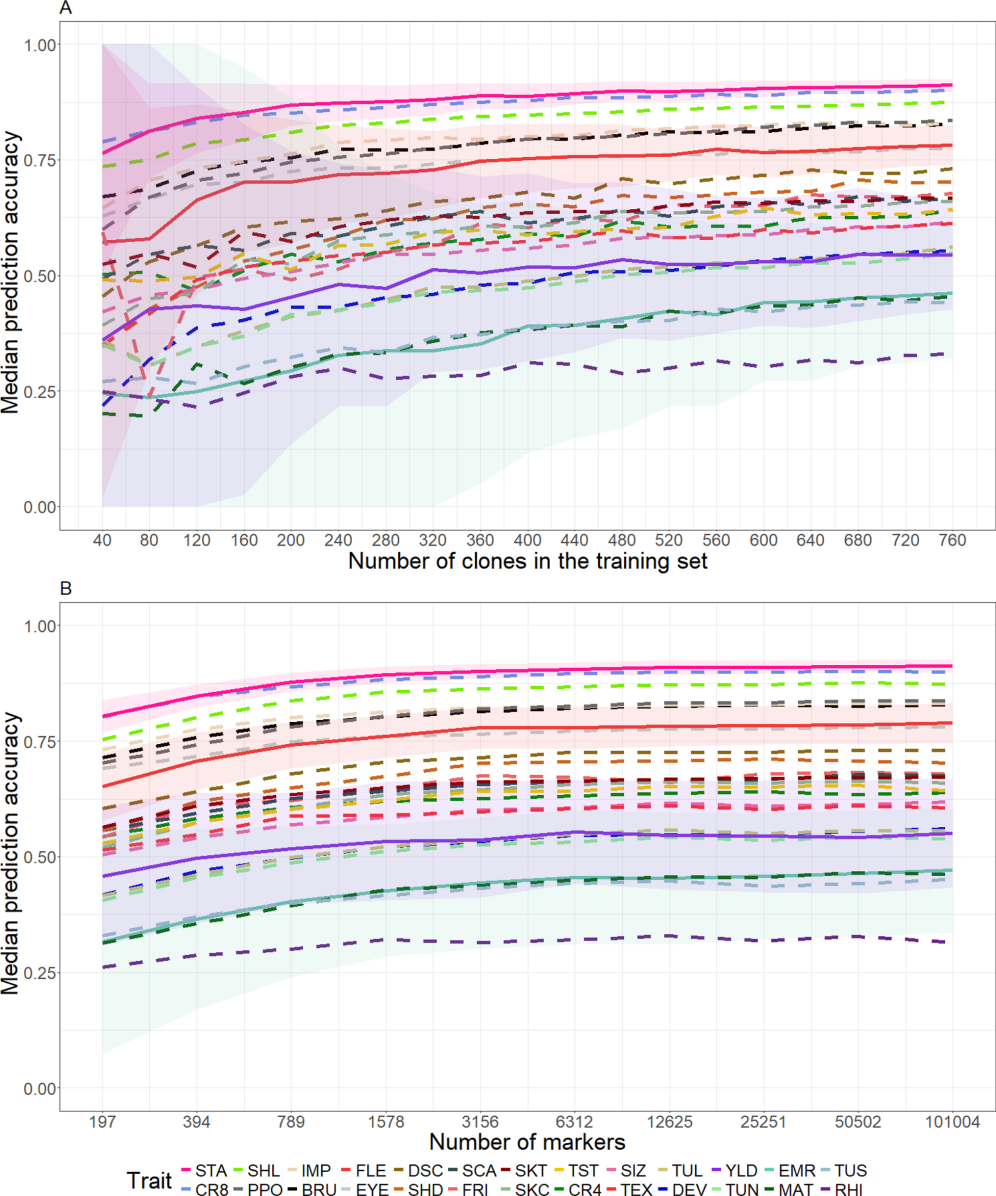
Median prediction accuracy (PA) of genomic predictions using different subsets of clones (A) and markers (B) of the complete data set for the 26 evaluated traits. The median PA was calculated across the fivefold cross-validation with 50 replications. Shaded backgrounds indicate the coefficient of variation (CoV) and are shown for the traits starch content (STA, pink), flesh color (FLE, orange), yield (YLD, purple), and emergence (EMR, green). For abbreviations of the traits, see Table 1

In a next step, the influences of the size of the TS (Fig. 3A) and the number of SNPs (Fig. 3B) on the PA were examined. The minimum size of the TS, which was needed to reach an optically defined plateau, depended on the trait (Fig. 3A). Traits with an overall lower PA needed in general a bigger TS to reach the plateau (approx. 480 clones) compared to traits with a higher PA, where a TS size of roughly 280 clones was sufficient to reach the plateau. Nevertheless, the ranking of the traits regarding the PA remained the same irrespective of the TS sizes. Across all traits, a bigger TS size led to a reduced coefficient of variation (CoV) of the PA. However, we observed smaller CoVs even for small TS sizes when the median PA was high.

When varying the number of SNPs used for the estimation of the genetic relationship matrix $\mathbf {G_a}$, the same trend of trait-specific differences regarding the minimum number of markers to reach a plateau can be observed as for the TS size (Fig. 3B). For traits with a high median PA, 2,000 SNPs were enough to reach the plateau, while traits with a lower median PA needed about 10,000 SNPs. Increasing the number of SNPs further only led to a marginal increase in PA. In addition to the PA, the number of SNPs also strongly impacted the CoV. The use of less than 2,000 SNPs to estimate the genetic relationship matrix yielded high CoVs for all traits. However, traits with high median PA showed again smaller CoVs than those with a low PA (Fig. 3B).

*Effect of the variability in the number of clones per segregating population:*

We analyzed the effect of the unbalancedness of the number of clones per segregating population by comparing a TS which was build using a balanced number of clones from each segregating population, an unbalanced number of clones from each population which represented the population size of the complete set by stratified sampling, and a completely random set of clones (Figure S4). For the first two scenarios, clones from smaller populations were either included completely or in an alternative scenario excluded. The mean of the median PAs across all traits was in the case of including clones from smaller populations 0.61 for the balanced setting, 0.65 for the unbalanced setting, and 0.64 for the random sampling. For the scenario, in which the clones from the smaller populations were excluded, the mean of the median PAs across all traits was 0.56, 0.59, and 0.58 for the balanced, unbalanced, and random setting, respectively. The ranking of the methods was trait dependent. The balanced number of clones per segregating population yielded across all traits mostly the smallest PA.

*Transferability of GP models across market segments and breeding companies:*

**Fig. 4:**
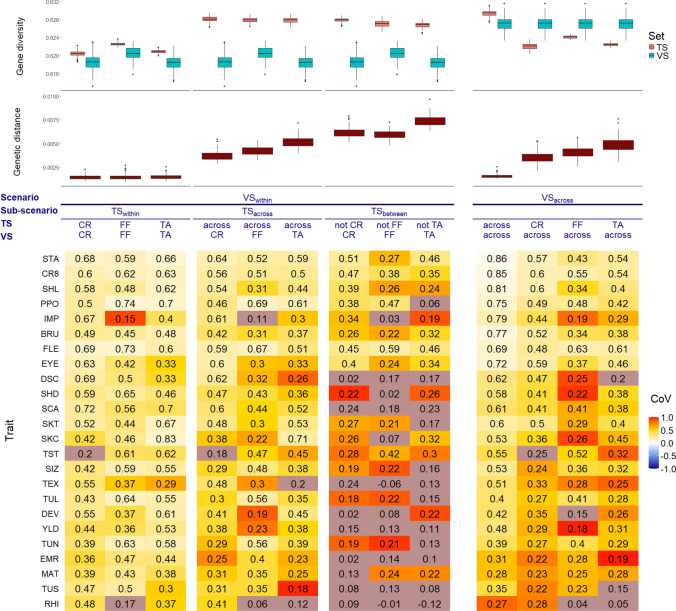
Heatmap of the median prediction accuracy (PA, numbers) and the respective coefficient of variation (CoV, colors) for prediction scenarios using clones from different market segments (TS size = 200 clones). The analyzed scenarios are described by their combination of clones from different market segments in the training set (TS) and validation set (VS). Analyzed market segments are crisps potato (CR), French fries potato (FF), and table potato (TA). In the sets where clones from more than one market segment are present, also clones from market segment starch production (ST) are present. Across designates a combination of clones from all market segments. CoVs higher than 1 or smaller than -1 are marked in brown. The boxplots above the heatmap represent the gene diversity ($\mathrm {H_e}$) of each TS and VS and the measures of genetic distance for each TS-VS combination for each scenario. For abbreviations of the traits, see Table 1

We evaluated two different prediction scenarios in our study. On the one side, the prediction of differences among the clones of one market segment ($\textrm{VS}_{\textrm{within}}$). In this situation, the highest PAs were observed after correcting for TS size in the $\textrm{TS}_{\textrm{within}}$ sub-scenario, i.e., where clones from the same market segment were part of the TS and the VS (Fig. 4). For some traits, the median PA within a specific market segment differed remarkably from the PA in the other market segments (e.g., IMP in FF or TST in CR). For these traits, also the CoV was much higher in the market segments with the deviating PA compared to the rest of the market segments. In general, the market segment which yielded the highest PA for the $\textrm{TS}_{\textrm{within}}$ market segment prediction varied noticeably among the traits. A wider TS, as it was the case in the $\textrm{TS}_{\textrm{across}}$ sub-scenario, resulted in slightly lower PA than the $\textrm{TS}_{\textrm{within}}$ sub-scenario. However, the trends observed across the traits regarding the median PA in the $\textrm{TS}_{\textrm{within}}$ market segment analysis were similar to those of the $\textrm{TS}_{\textrm{across}}$ market segment analysis. Predicting the GEBVs of the clones of one market segment by only using clones from other market segments in the TS further reduced the median PA across all traits and market segments even more. This $\textrm{TS}_{\textrm{between}}$ market segment sub-scenario also showed the highest variation in the PA across the CV runs. The measure of genetic differentiation $Q_{ST}$ ranged across all traits from 0 to 0.74 (Table 2). For some trait-market segment combinations, the predictions resulted in a negative PA, which were TEX in FF and RHI in FF and TA. Using the clones of one market segment to predict the clones of one other market segment in the $\textrm{TS}_{\mathrm{between-pairwise}}$ market segment prediction yielded comparable PAs to the $\textrm{TS}_{\textrm{between}}$ market segment sub-scenario (Figure S5).

Next to the before-described scenario, where the goal was to predict clones of a specific market segment, it might also be of interest how well clones of various market segments can be predicted. Therefore, we also analyzed the scenario $\textrm{VS}_{\textrm{across}}$, where the VS was built from clones across all market segments and the TS was on the other hand restricted to one market segment or also built across market segments (Fig. 4). Here, the highest PA was observed when using the broadest TS, i.e., a TS which was also built from clones across market segments ($\textrm{TS}_{\textrm{across}}$). In comparison with a TS made up across market segments, the PA was only about 2/3 when using a TS made up from one market segment.

In order to understand better the reasons for the observed pattern of PA, genetic distances between the TS and the VS and gene diversities within each TS and VS were calculated. In the case of a VS made up from one market segment ($\textrm{VS}_{\textrm{within}}$), the $\textrm{TS}_{\textrm{within}}$ market segment sub-scenario revealed the smallest genetic distances between the TS and the VS, while in the case of a $\textrm{VS}_{\textrm{across}}$ market segment prediction, the $\textrm{TS}_{\textrm{across}}$ showed the smallest genetic distance between TS and VS. Furthermore, we observed market segment specific differences in the genetic distance between the TS and the VS for the $\textrm{TS}_{\textrm{across}}$ - $\textrm{VS}_{\textrm{within}}$ market segment and $\textrm{TS}_{\textrm{between}}$ - $\textrm{VS}_{\textrm{within}}$ market segment analyses, where the $\textrm{TS}_{\textrm{across}}$ - $\textrm{VS}_{\textrm{within}}$ showed the smaller genetic distances between the TS and the VS compared to the $\textrm{TS}_{\textrm{between}}$ - $\textrm{VS}_{\textrm{within}}$ market segment sub-scenario. The highest genetic distance between the TS and the VS in the case of the $\textrm{VS}_{\textrm{across}}$ scenario was observed if the TS consisted of clones from the market segment TA.

Across all traits and sub-scenarios, the gene diversity was higher in the TS than in the VS when considering the scenario of market segment specific VS ($\textrm{VS}_{\textrm{within}}$). The gene diversity in the TS of the $\textrm{TS}_{\textrm{within}}$ market segment prediction ($\textrm{VS}_{\textrm{within}}$ - $\textrm{TS}_{\textrm{within}}$) was remarkably lower compared to the gene diversity of the TS from the other sub-scenarios. Within each sub-scenario, the differences in the gene diversity between the different market segments were low. For the case of a $\textrm{VS}_{\textrm{across}}$ market segment prediction, the gene diversity was higher in the VS than in the TS, except for the case where also the TS was built from clones across all market segments.

Across all sub-scenarios in the case of market segment specific VS ($\textrm{VS}_{\textrm{within}}$), we observed high market segment specific differences between the phenotypic variances. The phenotypic variance was across all traits mostly increased in the $\textrm{TS}_{\textrm{across}}$ and $\textrm{TS}_{\textrm{between}}$ market segment sub-scenario compared to the $\textrm{TS}_{\textrm{within}}$ market segment sub-scenario (Figure S6). Furthermore, for the second scenario of a $\textrm{VS}_{\textrm{across}}$ market segments, the VS showed a higher phenotypic variance compared to the TS.

In addition to the market segment subsets, we observed the effect of grouping clones within and across breeding companies. The general PA observed in these analyses was higher compared to the above-described market segment based analyses (Figure S7). However, the general trends of PA that were observed across the different subsets of breeding companies were similar to those of the different subsets of market segments (Figure S7 and Figure S8).

*Effect of truncation selection:*

**Fig. 5:**
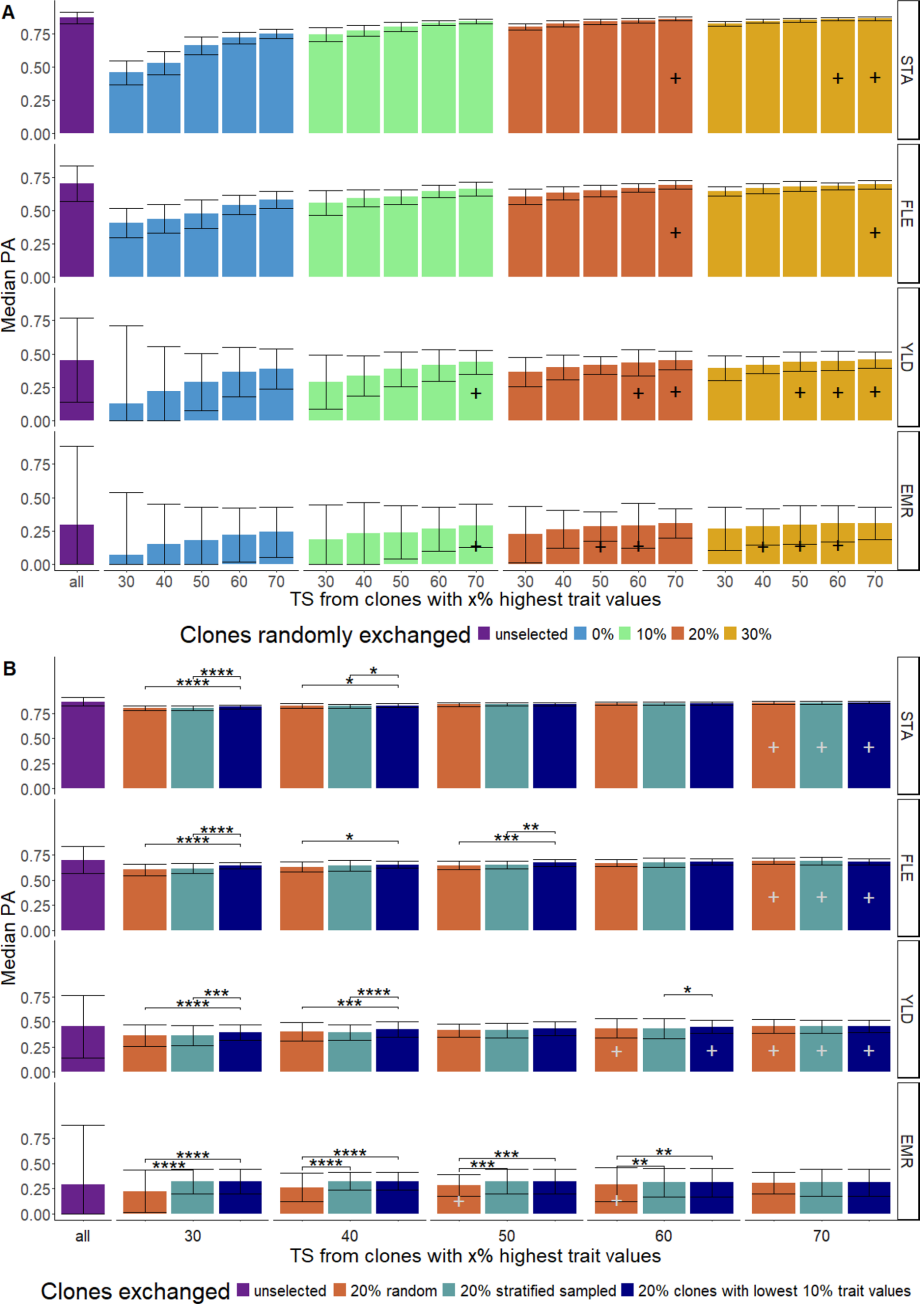
Median prediction accuracy (PA) and the respective coefficient of variation (CoV) for four representative traits when sampling the clones for the training set (TS) of the clones with the x% highest trait values, and additionally exchanging y% of clones in the TS with clones that do not belong to the clones with the x% highest trait values (A) and different methods to exchange 20% of the clones in the training set (B). In A, different amounts (y%) of clones were randomly exchanged from the TS, which was sampled from clones with the x% highest trait values, by clones which do not belong the clones with the x% highest trait values. In B, three different methods to exchange clones in the TS were used: i) randomly from the set of clones whose trait values were not in the x% highest values, ii) stratified from equally sized windows of the trait values, from all windows that were not included in the sampling of the TS, and iii) clones were sampled from the clones with the 10% lowest trait values. Plus signs indicate the recovery of the median PA of the unselected TS. For abbreviations of the traits, see Table 1

We examined the effect of trait-specific selection across the entire data set by taking only a subset of the clones with the x% highest trait values in the TS. We observed an almost linear decrease in the PA when restricting the TS to the clones with the highest trait values (Fig. 5A) despite a constant TS size. The CoV of the PA showed the inverse of this trend. The PA increased when randomly replacing 10, 20, or 30% of the clones in the TS by random clones that were not part of the clones with the x% highest trait values. For some cases, this inclusion led to a recovery of the original PA from the TS without selection. Including random clones that did not belong to the clones with the x% highest trait values also reduced the CoV and therefore yielded more stable results across all runs. While traits with a high PA predicted from a set of the 30% clones with the highest trait values yielded a medium PA and CoV (e.g., STA), this was not the case for traits with a lower median PA in the original scenario (e.g., YLD; EMR).

To evaluate the influence of the type of clone replacement, we compared different sampling methods for the exchange of 20% of clones in the TS with clones that did not belong to the set of clones with the x% highest trait values (Fig. 5 A and B). For all three cases in which the truncated TS was enriched by replacing clones of the TS, a higher PA was observed compared to the truncated TS alone (Fig. 5B). However, the increase was more pronounced the less random the replacement was organized. We observed, e.g., a stronger increase in the PA for the scenario where clones of the truncated TS were replaced by clones with the 10% lowest trait values compared to replacing them randomly. This difference in the sampling methods was particularly pronounced when selection was stronger. On the other hand, this effect was less important the lower the effect of the truncated selection was.

As breeders mostly do not select on one trait alone but on a combination of different traits, we also performed a selection on the best 50% of clones across all traits (Figure S9). Here, we found that the selection across multiple traits by the overall rank of a clone led to an increased PA compared to the selection on just the trait values of the trait of interest. However, also here an exchange of 20% of the clones in the TS with clones that belong to the clones with the 10% lowest ranks still improved the PA compared to using solely clones of the top 50% ranks as TS. However, this difference in the PA was less pronounced compared to the above-described individual trait analysis.

*Effect of selection on correlated traits in earlier generations:*

**Fig. 6:**
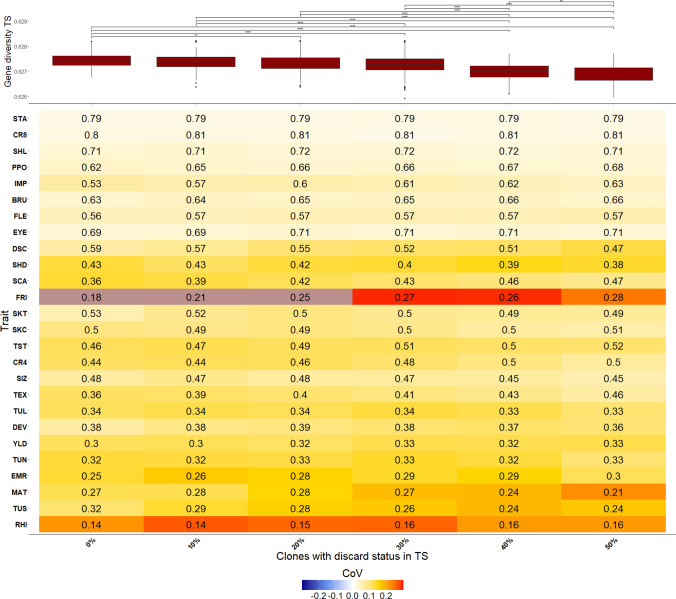
Heatmap of the median prediction accuracy (PA) and the respective coefficient of variation (CoV) for predictions where the training set of a size of 200 clones comprised an increasing amount of clones with discard status 1 (i.e., clones that would have been discarded in a commercial breeding program, but other clones of the same population would have been retained as A clones). Numbers indicate the median PA, while the colors show the CoV. CoV larger than 0.3 is marked in brown. The boxplot on the top represents the measures of gene diversity ($\mathrm {H_e}$) within each training set averaged over all replications. For abbreviations of the traits, see Table 1

Lastly, the effect of selection on correlated traits was investigated in our study by examining the PA using different TS that included varying proportions of clones that would have been discarded in the single hills stage in a commercial breeding program. Across all examined traits, no difference in the median PA was observed if clones with discard status 1 were included with up to 50% of the TS (Fig. 6). For some traits, e.g., IMP, TST, and TEX, an increase in the PA was observed, but for traits like, e.g., TUS, DSC, or SKT the reverse trend was observed. The CoV also showed no clear trend across all traits for the examined scenarios. Furthermore, the gene diversity decreased when adding clones with a discard status to the TS. This is presumably because the clones with discard status are more similar to each other than random clones.

To check whether the observed trends were caused by predicting across different market segments, which were selected by different criteria in the single hill stage, the effect of selection on correlated traits was also analyzed within the market segment CR alone (Figure S10). Here, we also found no noticeable difference in the median PA across the different traits when including clones with discard status or not.

## Discussion

### Prediction accuracy of key traits for potato breeding

We observed across all traits high PAs in this study when using all genotypes and markers (Fig. 2). This is presumably caused by the large number of clones in the TS, as well as the high number of SNPs compared to previous studies in potato (e.g., Stich & Van Inghelandt 2018; Endelman et al. 2018, ). The PA reported in our study for common scab symptoms (SCA) was high compared to the one found by Enciso-Rodriguez et al. (2018). Nevertheless, the results of our study for common scab symptoms as well as rhizoctonia symptoms (RHI) need to be treated with care as the disease symptoms were assessed in environments without artificial disease inoculation. This can lead to a low phenotypic differentiation among clones.

In our study, the genotype–environment interaction could be only estimated for the checks, as only these were repeated in the individual environments. However, as revealed in our companion study (Thelen et al. 2025), the genotype–environment interaction was in general trait specific and especially high for the traits emergence, skin type, foliage development, and general impression. We observed a clear tendency that traits where phenotypic variation was largely driven by genotypic variance compared to environmental as well as genotype–environment interaction variance, as found in our companion paper (Thelen et al. 2025), yielded the best PAs, e.g., STA, FLE, and SHL. In contrast, traits with a lower importance of genetic variance and, correspondingly, a higher importance of environmental variance led to lower PAs (e.g., MAT, YLD, and TUS), even though we considered these differences in the genetic complexity by comparing the PA, which includes *h*, and not prediction abilities, which would have been the correlation of the AEMs and the GEBVs. The high negative correlation of the PA of the different traits and the importance of genotype–environment interaction for the corresponding traits was in accordance with previous results (Wilson et al. 2021b). Furthermore, we found that traits with a high PA showed less dispersion of the PAs across the individual runs of the CV procedure. Therefore, for traits with higher PA, not only the accuracy was higher but also the precision, which makes them particularly attractive targets for GP approaches. This is especially relevant for traits whose assessment on the phenotypic level is laborious or requires a destruction of tubers, such as CR8, BRU, FRI, or DSC, so that currently these traits can only be used for selection in advanced stages.

One reason for the lower PA of some traits is presumably the way how the traits were assessed. We observed that traits with a stronger influence of environmental effects and lower heritability were rated on an ordinal scale. This classification might add some subjectivity and potential misclassification (Azevedo et al. 2024), although it was performed based on methods commonly used in the breeding programs, which should minimize the given subjectivity. Furthermore, the rating was carried out by only one person per block or environment, so at least a part of the remaining subjectivity should be covered in the calculation of the AEMs (Model 2) by the block- or environmental effect. However, this illustrates the potential of high-throughput phenotyping approaches, which allow a quantitative assessment of phenotypic properties and thereby might increase the PA.

An additional factor that potentially explains the differences in PA among the traits is the chosen GP model. GBLUP is known to work best for traits that are influenced by many small genetic effects (Onogi et al. 2015). Therefore, we have evaluated for some traits, which have been reported previously to be oligogenically inherited such as FLE, EYE (Van Eck 2007), or PPO (Werij et al. 2007; Urbany et al. 2011), the PA of the BayesA GP model that is particularly suitable for traits with major effect loci (Meuwissen et al. 2001). However, only a little improvement in the PAs was observed for these three traits using BayesA instead of GBLUP (increase of 0.02 to 0.06, data not shown), which is in accordance with the results of Stich & Van Inghelandt (2018) and Wilson et al. (2021b). Therefore, we think that an inadequacy of the chosen GP model is not the main reason for PA differences among traits.

The global as well as the pairwise $F_{st}$ values observed in our study (Table S3) suggested that the structure in our data set is considerably lower compared to that reported in other species (e.g., Akohoue et al. 2020; Windhausen et al. 2012). This was also supported by the PCA (Figures S2 and S3), where the PCs were made up from the SNPs that were also considered in the GBLUP analysis. Therefore, even if stronger population structure than presented here would be present, we do not expect from a methodological perspective that the conclusions made on the GBLUP model would change, if we used a prediction method that more directly accounted for population structure, e.g., by including the top PCs as covariates (Guo et al. 2014). Thus, for all following analyses, only the GBLUP model was used for computational reasons.

In our study, GP based on the GBLUP model with additive effects, as well as with additive and dominance effects, was examined. We found no improvement in the PA using the additive-dominance GBLUP compared to the additive GBLUP for all traits except FLE (Fig. 2). This is in agreement with earlier studies. Amadeu et al. (2020) found little differences in the PA comparing different modeling strategies, including additive and additive-dominance BLUP. Wilson et al. (2021b) and Endelman et al. (2018) found trait-specific differences for the importance of dominance effects, where the PA of most traits was not improved by adding dominance effects to the model. Stich & Van Inghelandt (2018) found for four out of seven traits an increase in the PA when modeling dominance effects. However, the traits examined in all these studies differ largely, making it difficult to conclude on the reason for the observed differences. Nevertheless, we can conclude that across all traits of our study, an additive model was sufficient to achieve high PAs and was therefore used for all further analyses in this study.

### Factors influencing the PA

The proper setup of TS is an important task in breeding programs starting to implement GP. Increasing the training set size led to higher PAs (Fig. 3A), which is in accordance with other studies in potato (Wilson et al. 2021a; Endelman et al. 2018). Nevertheless, TS sizes of around 400 clones led to almost the same PA and CoV as TS sizes of 760 clones. This is in contrast to, e.g., the results of Norman et al. (2018), who found in wheat a strong increase in the PA for up to 2,000 genotypes in the TS. This difference is presumably not due to differences in genetic complexity of the examined traits, as both studies examined traits of varying genetic complexity. Instead, the reason for smaller TS size requirements in our study compared to Norman et al. (2018) might be that in the latter a diversity panel was examined, while we studied clones from multiple segregating populations, which tends to reduce the genetic diversity. This means that in our study a smaller TS size was sufficient for an overall good PA, as more distant relationships might increase the noise and add bias to the prediction (Norman et al. 2018; Hickey et al. 2014).

In our study, the clones of the respective TS were chosen randomly. Wilson et al. (2021a) found a significant interaction between sampling method and sampling size. Thus, changing the sampling method from random sampling to analytical sampling may further decrease the minimal TS size for maximal PA and therefore reduce phenotyping costs. However, this aspect was not further examined in our study.

We found no increase in the median PA after passing a threshold of roughly 10,000 SNPs for prediction (Fig. 3B). Also, Sverrisdóttir et al. (2018) found that about 10,000 SNPs were sufficient to obtain reliable PAs, where SNPs were derived from genotyping-by-sequencing. The relatively small number of required SNPs could be resulting from the fact that the analyses in our study but also Sverrisdóttir et al. (2018) were performed on segregating populations. That means that the linkage disequilibrium is higher compared to that of diversity panels (e.g., Stich & Van Inghelandt 2018, ) and, thus, fewer SNPs are required in the former case to obtain high PA. In addition, we observed that traits with a higher PA required fewer SNPs to reach a plateau of the PA compared to traits with a lower PA (Fig. 3B). This is in agreement with the results of Aalborg et al. (2024), who also found a correlation between the PA and the required number of markers to reach a certain PA plateau.

To deal with missing data, mean imputation was used in our study. A more complex imputation method like for example the one implemented in beagle (Browning et al. 2018) is not possible. This is because linkage disequilibrium measures are mainly senseful to calculate only if the phase of the alleles is known (Slatkin 2008). However, this is not the case for most polyploid species (Gerard 2021). Due to the low rate of missing SNPs in our data set, we only expect marginal differences between scenarios of mean imputation and more sophisticated approaches and, thus, the simplest procedure was used in this manuscript.

In our analyses, the SNPs were chosen purely based on trait independent aspects such as minor allele frequency and missing data rate (Baig et al., in preparation). However, a trait-specifically chosen subset of SNPs might further decrease the number of required SNPs. This was shown for fry color by Byrne et al. (2020), who found a higher prediction ability for SNPs that were selected by genome-wide association scans of the trait of interest compared to random SNPs. These findings demonstrated that an overall reduction of the number of markers can further decrease the genotyping costs and therefore make GP an even more efficient procedure. However, as this selection needs to be performed for each of the many traits of potato, this might not lead to a dramatic reduction of the total number of markers. Furthermore, with today’s genotyping technologies, the number of genotyped loci is typically not the main limitation. Therefore, we did not examine this aspect further in our study.

The before described analyses were each build using a fivefold CV approach, i.e., the PA was calculated based on the same base population for the TS and VS, meaning they are not genetically independent of each other as the same alleles are shared and are not from a new breeding cycle. This has been shown before to inflate the PA (Werner et al. 2020) and might not reflect the real in-field situation, where each year a new breeding cycle is started. In the frame of our study, we are not able to assess the size of this effect as genetic material from additional breeding cycles is required.

In our study, the number of clones is largely unbalanced across segregating populations. This reflects the breeder’s reality. We tested in a sub-analysis the influence of the unbalanced number of clones per segregating population, comparing three sets of equal size: a balanced set across all segregating populations with the unbalanced set and a completely randomly sampled set (Figure S4). This analysis revealed a marginal effect of the unbalancedness on the PA.

### Transferability of prediction models among genetic groups

Potatoes from different market segments differ with respect to their profiles of trait values. Therefore, potato breeders often subdivide their genetic material into sub-groups that we designated in our study as market segments. Here, we compared the PA of predictions using combinations of clones from various market segments with those from only one market segment in the TS or VS. We considered two different perspectives. The first perspective was the case when breeders want to predict the performance of a set of clones from one market segment ($\textrm{VS}_{\textrm{within}}$) by using clones from the same or other market segments in the TS. We found that an addition of clones from other market segments ($\textrm{TS}_{\textrm{across}}$) to predict the clones of one market segment did not improve the PA (Fig. 4). In addition, we observed that the genetic distance between TS and VS increased in the $\textrm{TS}_{\textrm{across}}$ prediction sub-scenario compared to the $\textrm{TS}_{\textrm{within}}$ prediction sub-scenario. This is in accordance with findings from Lorenz & Smith (2015) in barley, who also found a decrease in the PA when adding more genetically distant inbreds. These findings illustrate that the clones from the same market segment are diverse enough to allow high PA and, on the other side, do not add noise due to high genetic distance (Norman et al. 2018; Hickey et al. 2014). This first point was supported by our analysis of the genetic differentiation ($Q_{ST}$, Table 2), which revealed that across all traits a high amount of genetic variance can be observed within the market segments compared to the complete genetic variance and, thus, the variance within the market segments seemed overall enough to reach a good PA.

A consequence of the strategy to rely on within market segment prediction could be that some breeding programs who cannot afford a TS for each market segment may instead perform predictions for market segments that are of lower priority to them on the basis of between market segment predictions. We observed that the $\textrm{TS}_{\textrm{between}}$ market segment prediction was only meaningful for traits that showed very high PA in the $\textrm{TS}_{\textrm{within}}$ market segment prediction, like STA, CR8, or SHL. However, especially for these traits, the variation among the different market segments was higher (Table 2), indicated by a positive correlation of the PA of the $\textrm{TS}_{\textrm{between}}$ market segment prediction (Fig. 4) and the $Q_{ST}$ value (Table 2). This is not intuitive, as this means that higher differences between the market segments did overall not decrease the PA but instead increased them. One explanation for this observation is that the genetic differences between the market segments might be due to the influence of some major genes on the phenotype. However, the polygenic background of these traits has to be caused by variation at the same loci in the different market segments. Otherwise it is not possible to explain why the prediction works well also in the presence of high $Q_{ST}$ values.

The low PA values in the $\textrm{TS}_{\textrm{between}}$ market segment prediction scenario for most traits can most likely not be improved by increasing the marker density as found by Hickey et al. (2014) and Norman et al. (2018). This is because the overall marker number in our study exceeded the marker numbers used in those studies. The low PA values might be explained by genetic differences between TS and VS (Table 2 and Fig. 4), meaning that different loci are responsible for the phenotypic variation in the different market segments. Also, different linkage disequilibrium blocks in the different market segments can explain the low PA values in the ($\textrm{TS}_{\textrm{between}}$) market segment prediction sub-scenario. In summary, this first scenario of the analysis ($\textrm{VS}_{\textrm{within}}$) showed that for a prediction of clones from one market segment it is best to completely rely on clones from the same market segment, as an addition of more genetically distant clones reduced the median PA.

However, one can also imagine that breeders might be interested in predicting the performance of clones of unknown market segments or even predict the assignment to a market segment. We found that this scenario, i.e., prediction of a $\textrm{VS}_{\textrm{across}}$ market segments, worked best when also the TS was built across market segments (Fig. 4). This was not surprising, as only in the case of a $\textrm{TS}_{\textrm{across}}$ the complete genetic diversity of the VS was also covered in the TS and, therefore, the genetic distance was small between TS and VS. However, we also found acceptable PA when the TS was built from clones of individual and especially the CR market segment. Therefore, we recommend breeders to broaden the TS when the goal is to predict across market segments so that a bigger part of the genetic diversity of the VS is covered in the TS, which improves the PA.

Collaborating breeding companies may decide to exploit a joint TS. Therefore, we evaluated the PA of breeding company specific TS and between breeding company predictions. Overall, we observed the same tendencies as described above for the market segment analysis (Figure S7). The higher PA in the breeding company analyses might be explained thereby that the clones split by market segments are a more strictly defined genetic group than the clones split by breeding companies. Thus, even though the clones from each breeding company vary genetically from each other, the clones of one breeding company reflect a broader genetic and phenotypic spectrum than clones of one market segment, leading to higher PAs across all sub-scenarios. This explanation is also underlined by the PCA (Figure S3), which showed that clusters per market segment were more clearly defined than clusters per breeding company.

In summary, this analysis showed that it is not effective for breeders to combine their breeding material for a joint TS when keeping the TS size constant. However, the addition of clones from other breeding companies, e.g., through a collaboration or through joint databases, is advantageous when this leads to an increased size of the TS (Fig. 3A).

### Effect of selection on the PA

The essence of breeding programs is that genotypes are discarded from the program based on assessed or predicted phenotypic values. This in turn influences which genotypes and/or data are available to serve as TS. Therefore, we assessed the effect of a simulated selection on the PA to provide a guideline for the setup of TS, especially if material from more advanced generations than A clones is used as TS.

We observed that a more intense selection led to lower PA values (Fig. 5A). This illustrates the importance of sufficient genotypic and phenotypic variation for obtaining high PAs. Similar findings have been reported before for maize (Zhao et al. 2012; Brandariz & Bernardo 2018).

In accordance with the results of Zhao et al. (2012), Brandariz & Bernardo (2018), and Michel et al. (2020), we observed that replacing clones of the truncated TS leads to increases in the PA. The inclusion of clones with lower trait values to the TS keeps a minimum amount of phenotypic variance and the resulting higher PA is therefore in accordance with the other findings in our study. However, our observation also suggests that it is sufficient and even preferable to include only clones that were sampled from the fraction with the lowest trait values when the clones in the TS are strictly selected for their high trait values (Fig. 5B). Our results suggest to include at least 20% of clones with low trait values to strongly selected TS, such as B or C clones, as this was the proportion of exchange that was needed to realize a PA that corresponds to the PA of the unselected TS (Fig. 5A). Our observation is in accordance with Michel et al. (2020) and Zhao et al. (2012).

These results based on individual trait selections are abstract, as potato breeders select for many traits simultaneously and, thus, the chance of only getting high-performing clones in the TS is low. This might lead to a TS, where also some clones with lower trait values were kept, as these clones performed well for the other traits of interest. We evaluated this aspect by a small example, using a TS comprising clones in the top 50%. But instead of sampling from the 50% highest trait values of one trait, we selected the top 50% based on the sum of the ranks across four example traits (STA, FLE, YLD, and EMR). Using the rank criterion instead of the single trait value as selection criterion increased the PA noticeably (Figure S9). This supports our explanation that the clones for each trait are more diverse when selecting for the overall rank. This difference between the PA of a selection based on the rank criterion *vs.* single trait values gets smaller when exchanging 20% of the TS with clones with lower trait values or ranks, as then both sets were more diverse. Nevertheless, also this result supports our above recommendation to include at least 20% of clones with low trait values to strongly selected TS, such as B or C clones.

The above-presented behavior of the PA can also be seen when choosing a trait combination relevant for breeding (Figure S11). We tested in a sub-analysis the trait combination STA 17% to 19%, CR8 high values, SHL low values, EYE high values, and BRU low values. However, we observed for the combination of these traits the same tendencies as described before for the combination of traits selected by the highest trait values. Therefore, we can conclude that the criteria for selection are not the important factor, but rather the fact that selection occurred in general, i.e., it is not important if selection is based on the highest trait values, lowest trait values, or based on a specific range. As long as breeding companies want to build a TS based on selected clones, it is preferable to add clones with non-preferable trait values to the TS to increase variability and thereby increase the PA.

Because of this considerable effect of selection on PA described above, we evaluated in the specific potato example if the performance of TS made up from A clones can be improved by including clones that would have been discarded in the single hill stage of commercial breeding programs. Across the examined traits, we found no increased PA when clones with discard status 1 were added to the TS (Fig. 6). This is in accordance with the observation that the gene diversity in the TS decreased when clones that would have been discarded in a breeding program were part of the TS. This finding can be explained by the lack of correlation between the traits assessed in the step prior to the A clone, i.e., the single hill stage, and the traits assessed in the later stages. This explanation is in agreement with the results of Thelen et al. (2025), who observed no mean differences between the genotypic variances of the sets including and excluding clones with discard status. We furthermore checked if the combination of different market segments in this analysis influenced the non-changing PA, as the different market segments were selected based on different thresholds and relative weights of the different traits. However, the same trend was observed for the analyses across market segments and within a market segment. This observation suggests that random sets of A clones are appropriate as TS and show enough genotypic and phenotypic diversity, and it is not necessary to include clones with a discard status from the single hills stage into the TS to increase diversity and correspondingly PA.

### Conclusions from a breeding point of view

Our analyses revealed overall high PAs, suggesting the relevance of GP for potato breeding. We found no improvement in the PA using the additive-dominance GBLUP compared to the additive GBLUP for all traits except one, suggesting the possibility to generate fast progress in many traits. Our results indicated that TS of about 400 diverse clones is sufficient to result in close to the maximal PA when considering segregating populations. Similarly, about 10,000 markers are sufficient to result in almost maximal PA for such material. We found that an addition of clones from other market segments to predict the clones of one market segment did not improve the PA compared to prediction within one market segment alone. A prediction between different market segments was in our study only meaningful for traits that showed a high PA in the within market segment prediction. Furthermore, we found that it is sufficient if breeding companies use their own genetic material to predict the breeding values of untested clones. Further, it is not necessary to include in the TS clones from the single hills stage that would normally have been discarded. Our analyses suggested that if genetic material that underwent strong phenotypic selection, such as B, C, or D clones, is used as TS, about 20% of clones with the opposite values than the selected clones should be included, to recover the original prediction accuracy of an unselected TS.

**Table 1:** Abbreviations and units for the evaluated traits considered in our study

Abbreviation	Trait	Unit	$h^{2}$	Method
BRU *	Susceptibility to bruising	%	0.89	5 kg tuber sample [1]
CR4 *	Crisps color after storage at 4$^\circ$C	1–9	0.79	1 = bad quality (very dark), 9 = good quality (no discoloration) [2]
CR8 *	Crisps color after storage at 8$^\circ$C	1–9	0.89	1 = bad quality (very dark), 9 = good quality (no discoloration) [2]
DEV	Foliage development	1–9	0.67	1 = not grown to very weak development, 9 = superior / extraordinary growth [2]
DSC *	After cooking discoloration	1–9	0.59	1 = very dark, 9 = no discoloration [2]
EMR	Emergence	1–9	0.74	rate of emergence from the soil, 1 = very poor, 9 = very good [2]
EYE	Eye depth	1–9	0.86	1 = very deep, 9 = very flat [2]
FLE	Flesh color	1–9	0.89	1 = white, 9 = blue/purple [1]
FRI *	French fry color	1–9	0.80	1 = bad quality (very dark), 9 = good quality (no discoloration) [2]
IMP	General impression	1–9	0.68	1 = deficiencies, 9 = very good [2]
MAT	Foliage maturity	1–9	0.84	1 = still flowering, 9 = dead, relative to checks [2]
PPO	Polyphenol oxidase activity	1–9	0.90	tuber flesh after DL-DOPA incubation, 1 = no color change, 9 = very dark coloration
RHI	Rhizoctonia symptoms	1–9	0.57	1 = >90 % infested, 9 = no symptoms
SCA	Common scab symptoms	1–9	0.45	1 = >90 % infested, 9 = no symptoms , 2-8 = up to 90, 60, 30, 20 10, 5, 2 % infected, respectively
SHD	Tuber shape diagonally	1–9	0.48	1 = round, 9 = flat
SHL	Tuber shape longitudinally	1–9	0.93	1 = round, 9 = long [2]
SIZ	Tuber size	1–9	0.82	1 = very small, 9 = very big [2]
SKC	Skin color	1–9	0.89	1 = cream, 9 = blue/purple [2]
SKT	Skin type	1-4	0.83	1 = smooth, 4 = cracked, 5 = russet [1]
STA	Starch content	%	0.95	under water weight measurement with automatic starch scale [1]
TEX *	Texture after cooking	1–9	0.50	1 = tuber falls completely apart, 9 = tuber stays tightly together [1]
TST *	Taste	1–9	0.33	1 = very strong deficits (e.g., bitter), 9 = nice potato taste [1]
TUL *	Proportion of large tubers >65 mm	%	0.88	weighing after grading
TUN *	Proportion of normal tubers 35-65 mm	%	0.85	weighing after grading
TUS *	Proportion of small tubers < 35 mm	%	0.86	weighing after grading
YLD	Total tuber yield	kg	0.78	normalized to a 16 plant plot

The traits are sorted alphabetically

$*$ only assessed in years 2020 and 2021

[1] Bundessortenamt (2019)

[2] Tiemens-Hulscher et al. (2013)

**Table 2:** Genetic variance between ($\sigma _{m}^{2}$) and within ($\sigma _{m(c)}^{2}$) market segments and the resulting degree of genetic differentiation $Q_{ST}$

	$\sigma _{m}^{2}$	$\sigma _{m(c)}^{2}$	$Q_{ST}$
STA	5.06	1.76	0.74
CR8	0.89	0.49	0.65
SHL	1.17	0.57	0.67
PPO	1.81	2.02	0.47
IMP	0.10	0.15	0.39
BRU	438.38	241.19	0.65
FLE	0.16	0.40	0.29
EYE	0.41	0.39	0.51
DSC	0.21	0.25	0.45
SHD	0.02	0.29	0.07
SCA	0.00	0.12	0.00
SKT	0.08	0.23	0.26
SKC	0.04	0.51	0.07
TST	0.06	0.09	0.38
SIZ	0.08	0.30	0.22
TEX	0.08	0.22	0.27
TUL	0.56	194.21	0.00
DEV	0.02	0.27	0.07
YLD	1.58	9.42	0.14
TUN	0.00	138.90	0.00
EMR	0.03	0.49	0.06
MAT	0.04	0.85	0.05
TUS	0.37	5.62	0.06
RHI	0.00	0.26	0.01

For abbreviations of the traits, see Table 1

## Key message

Genomic prediction in potato works best within market segments. Adding 20% low-trait clones to a high-trait-selected training set restores accuracy to that of an phenotypically unselected set.

## Supplementary Information

Below is the link to the electronic supplementary material.

Supplementary file 1 (pdf 1126 KB)

## Data Availability

The original data sets generated and/or analyzed in the current study are not publicly available due to the material being part of the company secrets of SaKa Pflanzenzucht GmbH & Co. KG, Nordring-Kartoffelzucht- und Vermehrungs- GmbH & Co. KG, and EUROPLANT Innovation GmbH & Co. KG. However, the data are available in encoded form from the corresponding author upon reasonable request.
