# Supplementary material for: Transferability of genomic prediction models across market segments in potato and the effect of selection
Source: Theor Appl Genet. 2025 Aug 20;138(9):219. doi: 10.1007/s00122-025-05004-9 (PMC12367938; doi:10.1007/s00122-025-05004-9)
Supplement: Supplementary file 1 — (pdf 1126 KB) [file 122_2025_5004_MOESM1_ESM.pdf]

SUPPLEMENTARY MATERIAL

Table S1. Selection scheme for the preselection in the single hills stage, for each market segment. PVY = Potato virus Y. For other abbreviations of the traits see Table 1.

TA		FF		CR		ST	
Trait	Weight/Priority	Trait	Weight/Priority	Trait	Weight/Priority	Trait	Weight/Priority
SKT	1	SHL	1	STA	1	STA	1
SHL	2	SIZ	2	SHD	3	SIZ	2
SHD	3	SCA	3	SCA	3	SCA	3
EYE	3	SHD	4	EYE	4	SHL	4
SCA	3	EYE	4	SHL	4	SHD	4
SKC	4	FLE	4	PVY	2	PVY	2
PVY	2	PVY	2				

Table S2. Environments (*i.e.* year-location combinations) that were used in our experiment and their respective properties. Each entry was replicated once per environment of the donor breeding company (five times in total), and each check was replicated eight times within each environment.

Environment	No. of entries	No. of populations	No. of blocks	No. of plants per plot
Europlant 2019 Kaltenberg	299	46	4	10
Europlant 2020 Kaltenberg	297	47	4	16
Europlant 2020 Böhlendorf	287	46	2	16
Europlant 2021 Kaltenberg	300	47	4	16
Europlant 2021 Böhlendorf	300	47	1	16
Norika 2019 Groß Lüsewitz	300	17	2	9
Norika 2020 Groß Lüsewitz	300	17	4	18
Norika 2020 Mehringen	300	17	3	20
Norika 2021 Groß Lüsewitz	297	17	4	18
Norika 2021 Mehringen	300	17	2	20
SaKa 2019 Windeby	458	107	8	10
SaKa 2020 Windeby	387	99	8	16
SaKa 2020 Gransebieth	387	99	8	16
SaKa 2021 Windeby	387	99	8	16
SaKa 2021 Gransebieth	387	99	8	16

Table S3. Pairwise fixation index F_{ST} for a cluster of the four market segments table potato (TA), crisps potato (CR), starch potato (ST), ans French fry potato (FF). Global F_{st}= 0.01.

	TA	CR	ST	FF
TA				
CR	0.02			
ST	0.02	0.02		
FF	0.01	0.01	0.02	

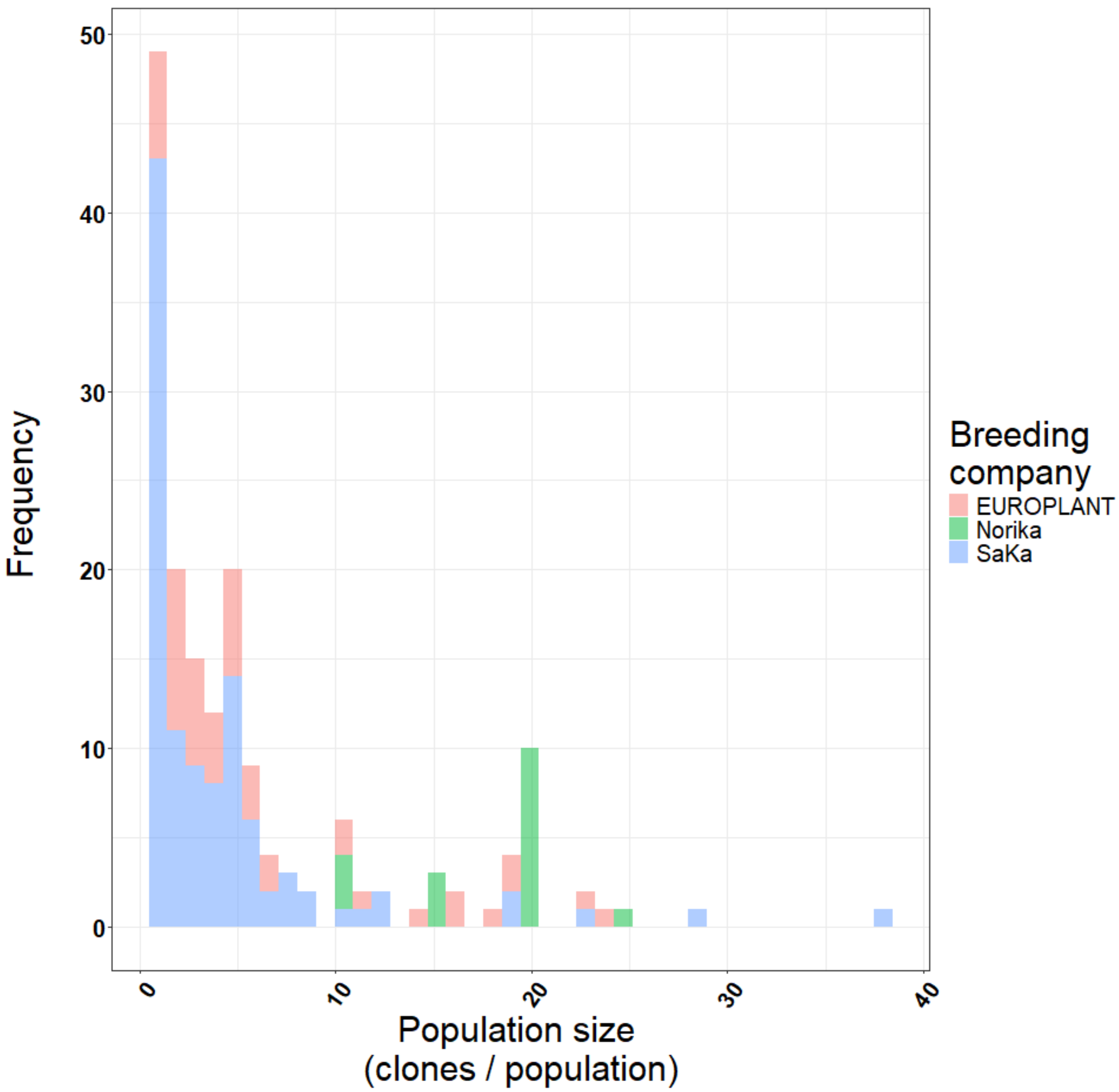

Fig. S1. Distribution of the sizes of the populations across breeders.

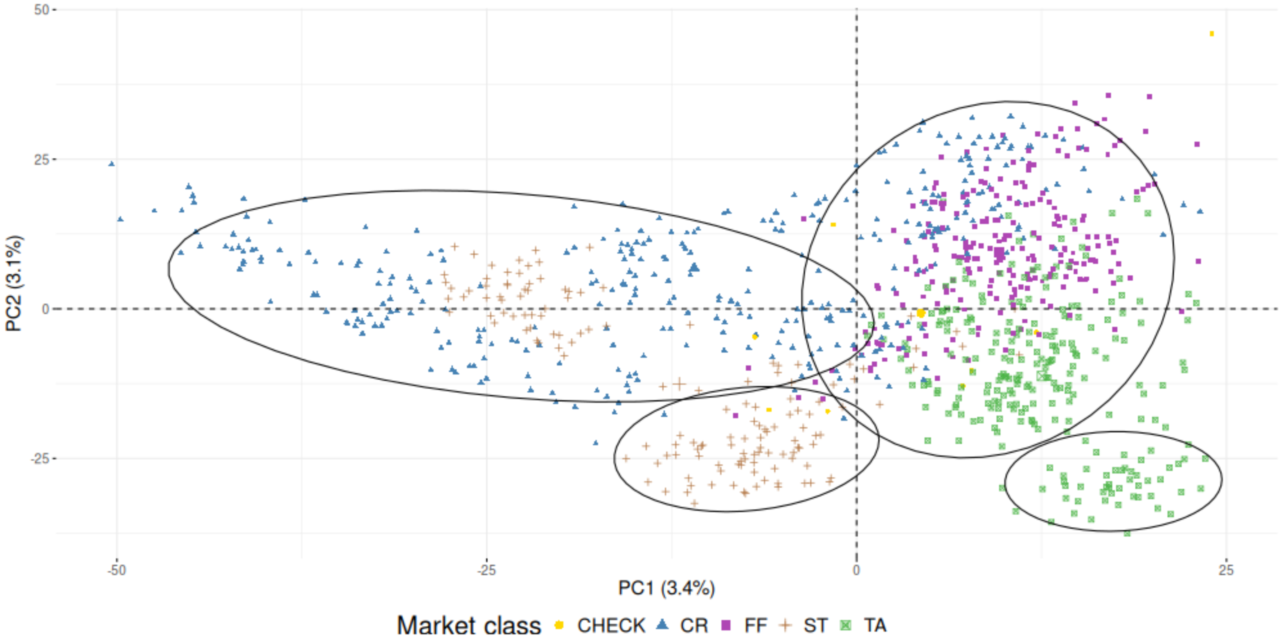

Fig. S2. Principal component analysis (PCA) of the 988 clones based on their marker data of principal component 1 (PC1) and principal component 2 (PC2). Numbers in parentheses refer to the proportion of variance explained by the principal component. Clones are colored by their respective market segments, which are crisps potato (CR), French fry potato (FF), starch potato (ST) and table potato (TA).

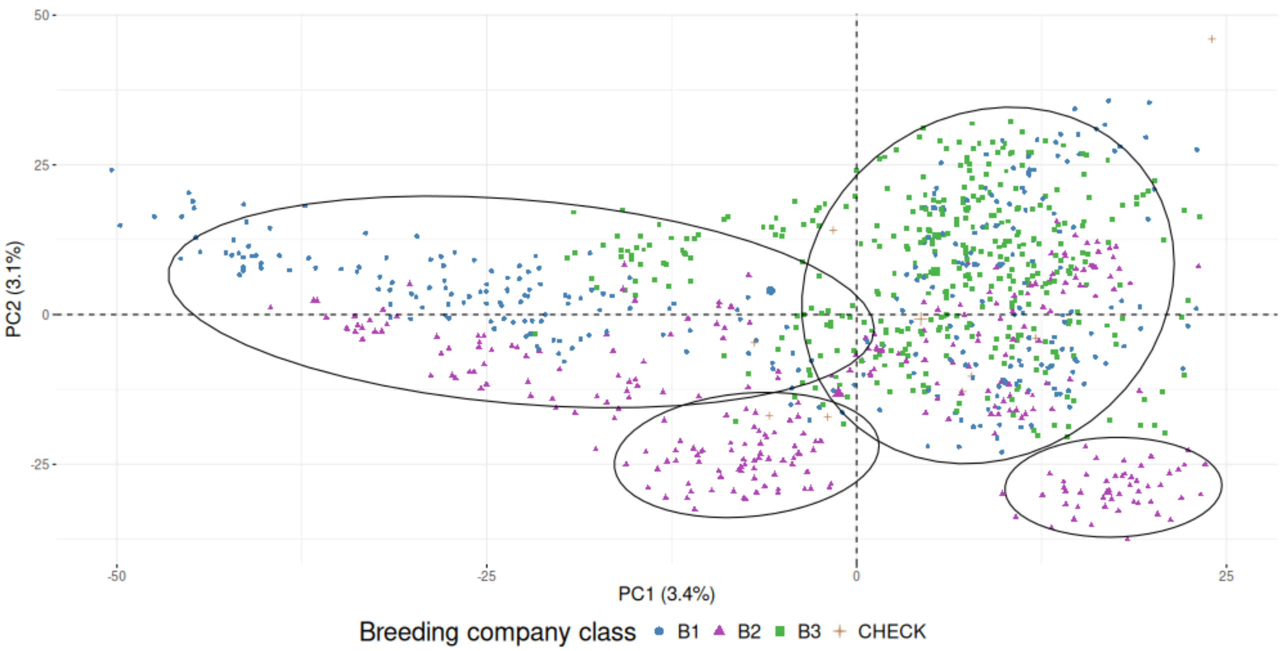

Fig. S3. Principal component analysis (PCA) of the 988 clones based on their marker data of principal component 1 (PC1) and principal component 2 (PC2). Numbers in parentheses refer to the proportion of variance explained by the principal component. Clones are colored by their breeding company (B1 to B3).

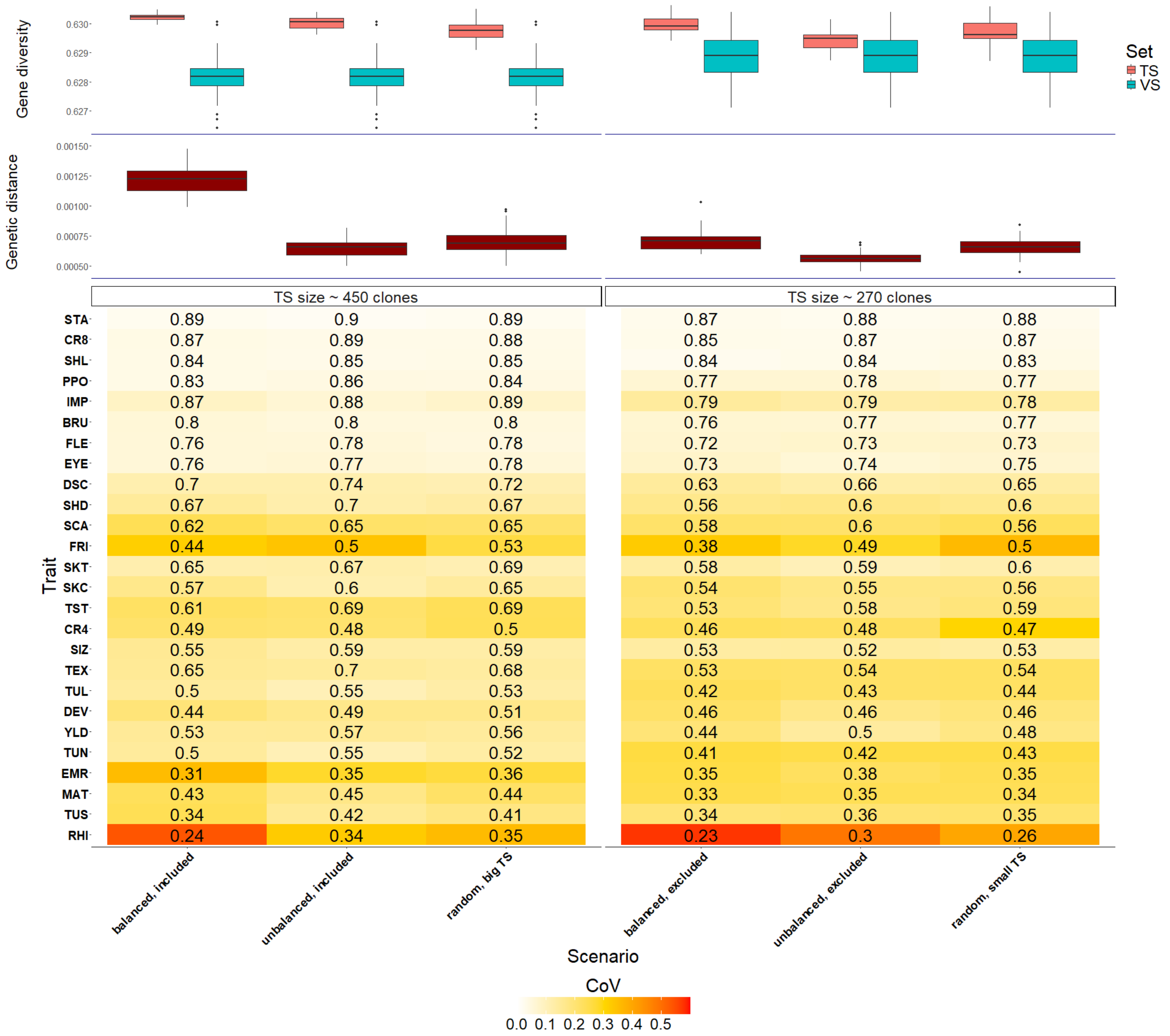

Fig. S4. Heatmap of the median prediction accuracy (PA) and the respective coefficient of variation (CoV) for predictions using different levels of unbalancedness of population sizes. For the balanced scenario, four clones were taken from each population. Populations with less than four clones were either included or excluded. For the unbalanced scenario, clones were sampled stratified in the same proportion as they occur in the complete set, but the training set (TS) had comparable sizes to that of the balanced scenario. In the random sampling scenario, clones were randomly picked across the corresponding populations. For abbreviations of the traits see Table 1.

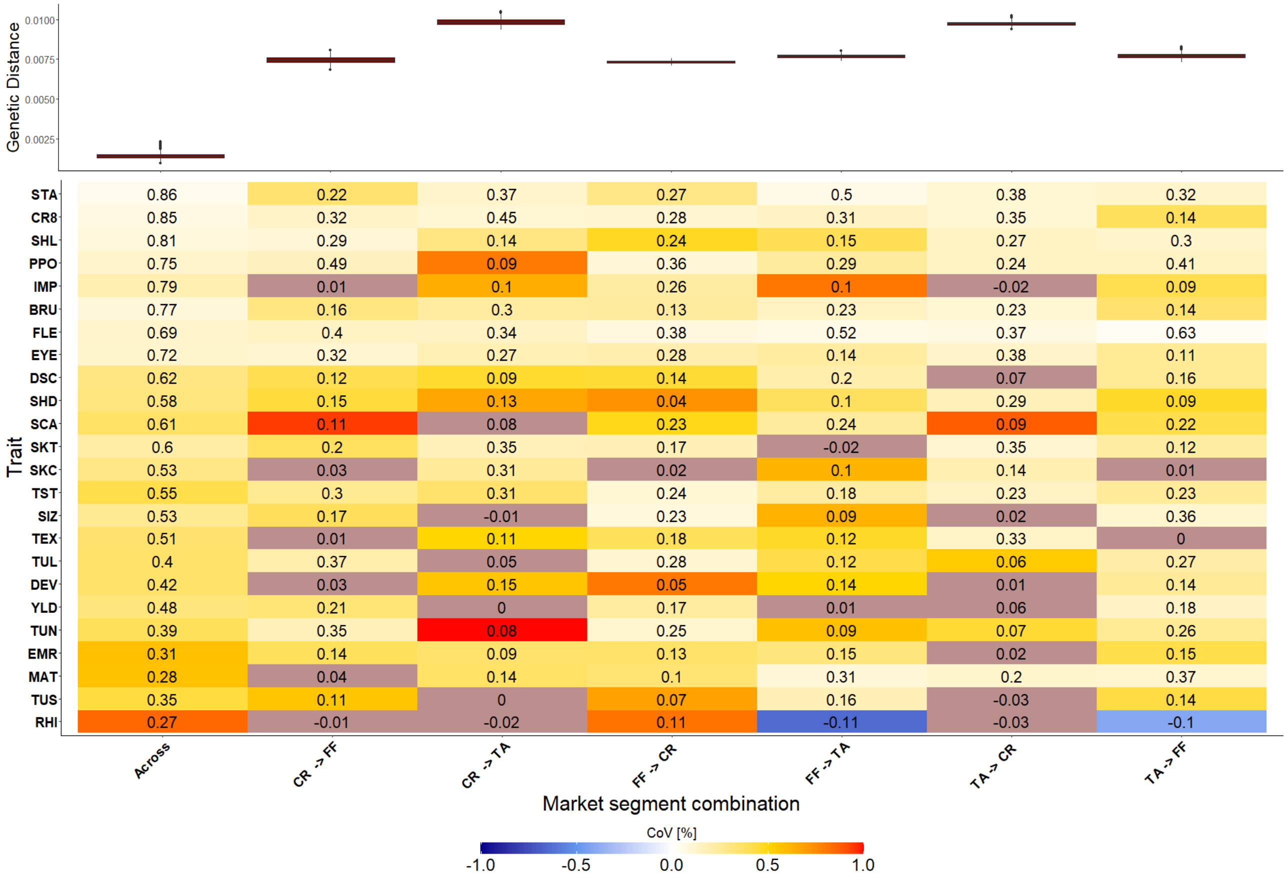

Trait	Across	CR -> FF	CR -> TA	FF -> CR	FF -> TA	TA -> CR	TA -> FF
STA	0.86	0.22	0.37	0.27	0.5	0.38	0.32
CR8	0.85	0.32	0.45	0.28	0.31	0.35	0.14
SHL	0.81	0.29	0.14	0.24	0.15	0.27	0.3
PPO	0.75	0.49	0.09	0.36	0.29	0.24	0.41
IMP	0.79	0.01	0.1	0.26	0.1	-0.02	0.09
BRU	0.77	0.16	0.3	0.13	0.23	0.23	0.14
FLE	0.69	0.4	0.34	0.38	0.52	0.37	0.63
EYE	0.72	0.32	0.27	0.28	0.14	0.38	0.11
DSC	0.62	0.12	0.09	0.14	0.2	0.07	0.16
SHD	0.58	0.15	0.13	0.04	0.1	0.29	0.09
SCA	0.61	0.11	0.08	0.23	0.24	0.09	0.22
SKT	0.6	0.2	0.35	0.17	-0.02	0.35	0.12
SKC	0.53	0.03	0.31	0.02	0.1	0.14	0.01
TST	0.55	0.3	0.31	0.24	0.18	0.23	0.23
SIZ	0.53	0.17	-0.01	0.23	0.09	0.02	0.36
TEX	0.51	0.01	0.11	0.18	0.12	0.33	0
TUL	0.4	0.37	0.05	0.28	0.12	0.06	0.27
DEV	0.42	0.03	0.15	0.05	0.14	0.01	0.14
YLD	0.48	0.21	0	0.17	0.01	0.06	0.18
TUN	0.39	0.35	0.08	0.25	0.09	0.07	0.26
EMR	0.31	0.14	0.09	0.13	0.15	0.02	0.15
MAT	0.28	0.04	0.14	0.1	0.31	0.2	0.37
TUS	0.35	0.11	0	0.07	0.16	-0.03	0.14
RHI	0.27	-0.01	-0.02	0.11	-0.11	-0.03	-0.1

Fig. S5. Heatmap of the median prediction accuracy (PA) and the respective coefficient of variation (CoV) for predictions of one market segment using another market segment (training set size = 200 clones). The first market segment describes the training set, and the second market segment describes the validation set. Across describes a scenario where clones from all market segments were used in the training set as well as in the validation set. Numbers indicate the median PA, while the colors show the CoV. CoVs higher than 1 or smaller than -1 are marked in brown. The above shown boxplot represents the measures of genetic distance for each training set-validation set combination. For abbreviations of the traits see Table 1.

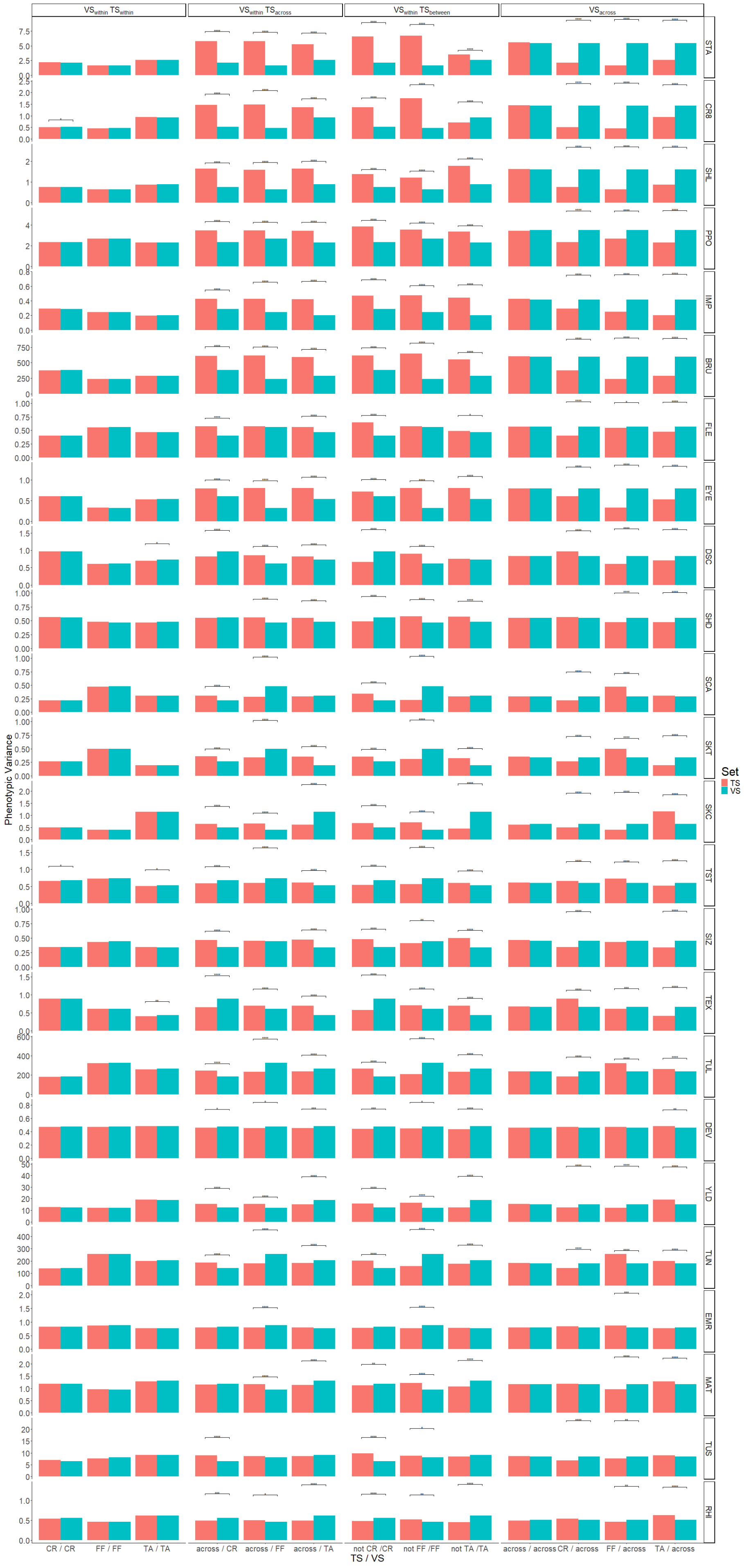

Fig. S6. Mean phenotypic variances of the clones in the training set (TS) and validation set (VS) across all runs for each scenario of the market segment analysis. For abbreviations of the traits, market segments, and scenarios see Figure 4.

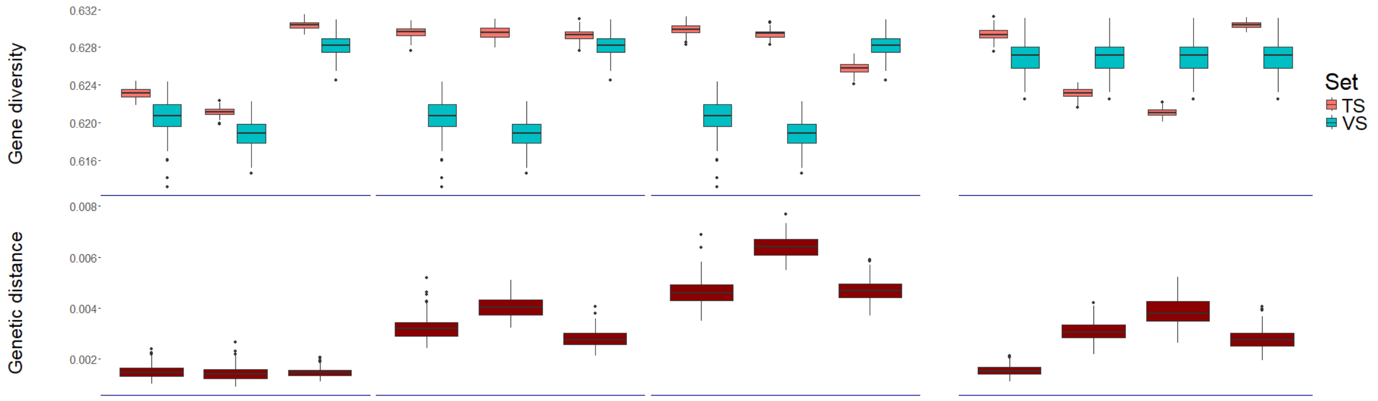

Scenario	VS_{within}									VS_{across}			
Sub-scenario	TS_{within}			TS_{across}			$TS_{between}$						
TS	B1	B2	B3	across	across	across	not B1	not B2	not B3	across	B1	B2	B3
VS	B1	B2	B3	B1	B2	B3	B1	B2	B3	across	across	across	across
STA	0.94	0.9	0.79	0.91	0.86	0.73	0.85	0.71	0.59	0.87	0.81	0.69	0.72
CR8	0.92	0.87	0.84	0.88	0.84	0.81	0.82	0.77	0.72	0.86	0.8	0.7	0.78
SHL	0.81	0.9	0.85	0.79	0.85	0.81	0.63	0.66	0.56	0.81	0.69	0.57	0.72
PPO	0.69	0.89	0.53	0.6	0.87	0.47	0.51	0.7	0.36	0.74	0.5	0.71	0.54
IMP	0.61	0.89	0.37	0.36	0.82	0.23	0.21	0.23	0.17	0.76	0.2	0.63	0.26
BRU	0.83	0.81	0.76	0.79	0.75	0.69	0.7	0.63	0.36	0.75	0.63	0.6	0.59
FLE	0.75	0.81	0.72	0.7	0.78	0.66	0.67	0.67	0.58	0.7	0.61	0.62	0.68
EYE	0.62	0.85	0.79	0.59	0.8	0.75	0.49	0.69	0.59	0.73	0.64	0.57	0.62
DSC	0.72	0.54	0.77	0.59	0.2	0.71	0.41	0.08	0.2	0.63	0.41	-0.02	0.62
SHD	0.65	0.56	0.43	0.56	0.44	0.38	0.32	0.25	0.18	0.58	0.38	0.39	0.35
SCA	0.44		0.41	0.34		0.26	0.15		0.04	0.57	0.27		0.29
SKT	0.76	0.62	0.58	0.69	0.51	0.53	0.61	0.41	0.38	0.61	0.54	0.33	0.53
SKC	0.78	0.64	0.47	0.7	0.46	0.31	0.43	0.33	0.17	0.54	0.46	0.29	0.35
TST	1.01	0.79	0.36	0.85	0.66	0.35	0.67	0.51	0.37	0.53	0.44	0.5	0.45
SIZ	0.67	0.5	0.52	0.57	0.43	0.39	0.41	0.28	0.2	0.49	0.42	0.4	0.35
TEX	0.67	0.71	0.37	0.53	0.61	0.33	0.37	0.4	0.29	0.55	0.38	0.4	0.38
TUL	0.59	0.41	0.42	0.49	0.32	0.29	0.24	0.24	0.14	0.4	0.37	0.29	0.32
DEV	0.48	0.41	0.49	0.28	0.23	0.43	0.09	0.06	0.15	0.42	0.16	0.16	0.32
YLD	0.55	0.71	0.37	0.47	0.64	0.29	0.34	0.35	0.15	0.45	0.38	0.36	0.32
TUN	0.55	0.41	0.4	0.43	0.32	0.27	0.23	0.22	0.14	0.4	0.35	0.29	0.29
EMR	0.35	0.31	0.48	0.24	0.17	0.38	0.2	0.03	0.12	0.29	0.13	0.19	0.24
MAT	0.53	0.43	0.27	0.37	0.4	0.22	0.17	0.22	0.17	0.3	0.24	0.3	0.2
TUS	0.55	0.41	0.36	0.41	0.28	0.2	0.15	0.08	0.08	0.3	0.25	0.24	0.2
RHI	0.29	0.59	0.2	0.04	0.43	0.08	-0.11	0.09	-0.01	0.27	-0.04	0.26	0.05

Trait

CoV: 1.0, 0.5, 0.0, -0.5, -1.0

Fig. S7. Heatmap of the median prediction accuracy (PA, numbers) and the respective coefficient of variation (CoV, colors) for prediction scenarios using clones of different breeding companies (B1 to B3, training set size = 200 clones). The analyzed scenarios are described by their combination of clones from different breeding companies in the training set (TS) and validation set (VS). Across designates a combination of clones from all market segments. CoVs higher than 1 or smaller than -1 are marked in brown. The boxplots above the heatmap represent the gene diversity of each TS and VS and the measures of genetic distance for each TS-VS combination for each scenario. For abbreviations of the traits see Table 1.

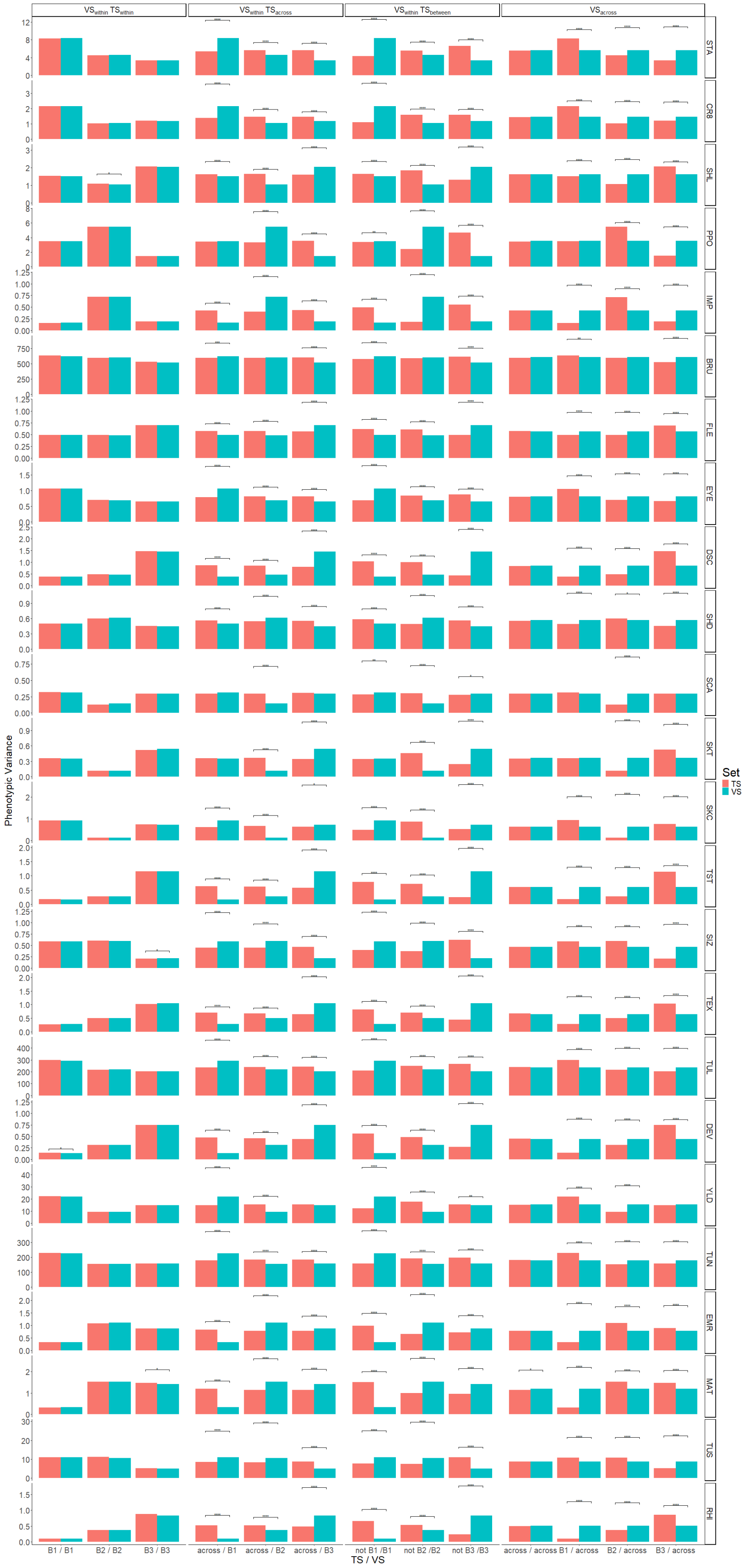

Fig. S8. Mean phenotypic variances of the clones in the training set (TS) and validation set (VS) across all runs for each scenario of the breeding company analysis. For abbreviations of traits and scenarios see Figure S7.

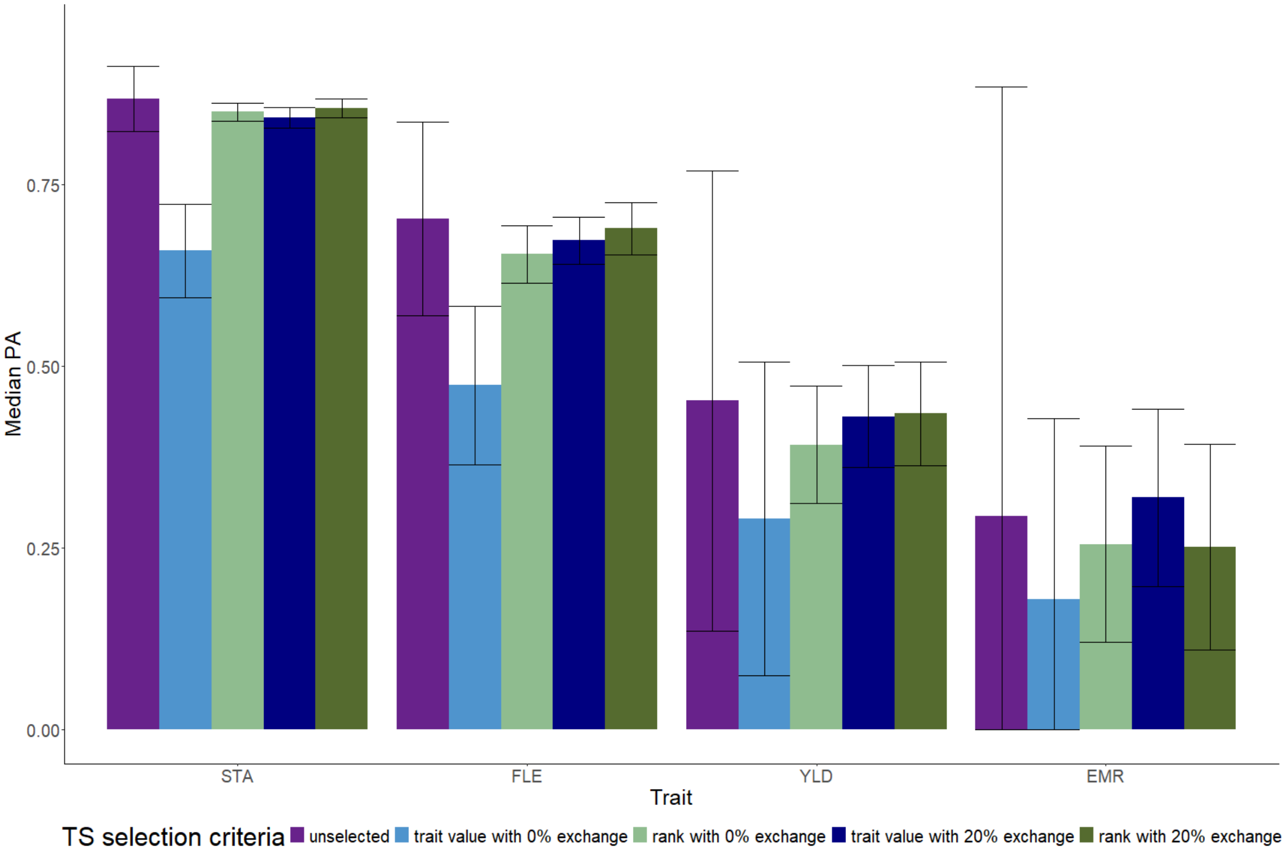

Fig. S9. Median prediction accuracy (PA) and the respective coefficient of variation (CoV) for four representative traits when sampling the clones for the training set (TS) of the clones with 50% highest trait values or ranks and additionally exchanging 0 or 20% of clones in the TS with clones that belong to the clones with 10% lowest trait values or ranks. Here, rank defines the combined rank as the sum of single ranks for all traits, while the trait values were trait-specific. For abbreviations of the traits see Table 1.

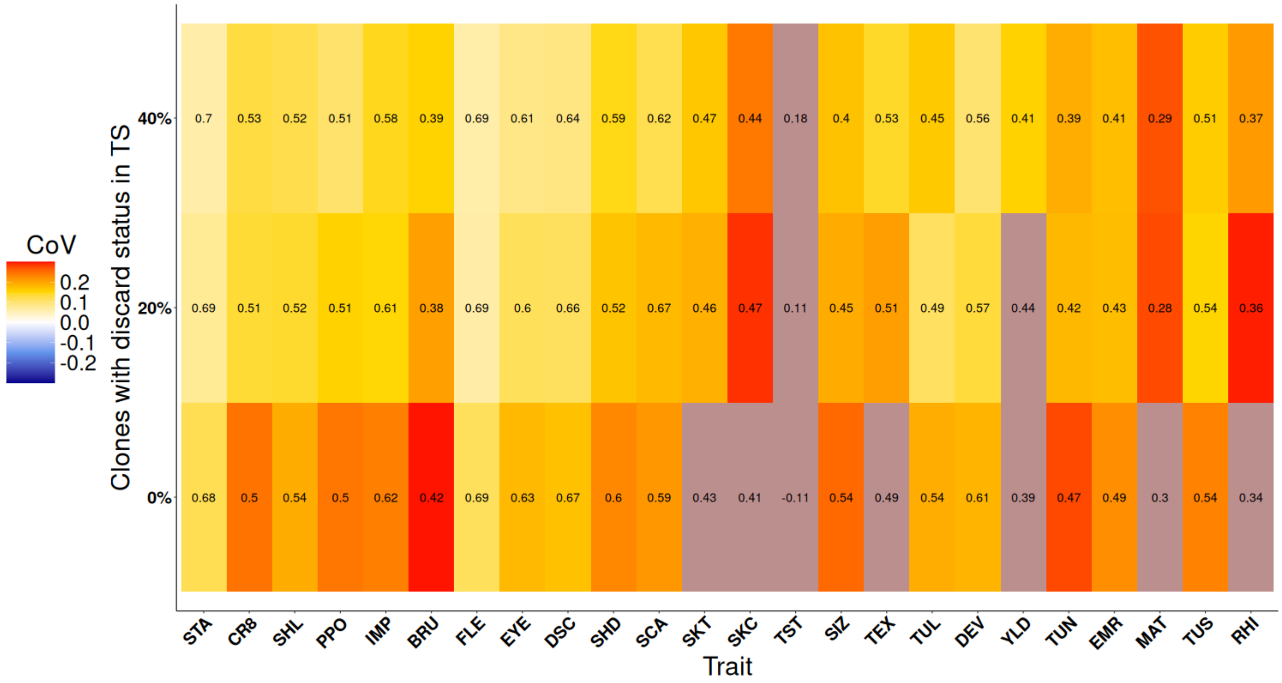

Fig. S10. Heatmap of the median prediction accuracy (PA) and the respective coefficient of variation (CoV) for predictions where the training set was exclusively from clones that belong to the market segment crisps potato and had an increasing amount of clones with discard status 1 (*i.e.* clones that would have been discarded in a commercial breeding program, but other clones of the same population would have been retained as A clones). Numbers indicate the median PA, while the colors show the CoV. TS size = 188 clones. For abbreviations of the traits see Table 1.

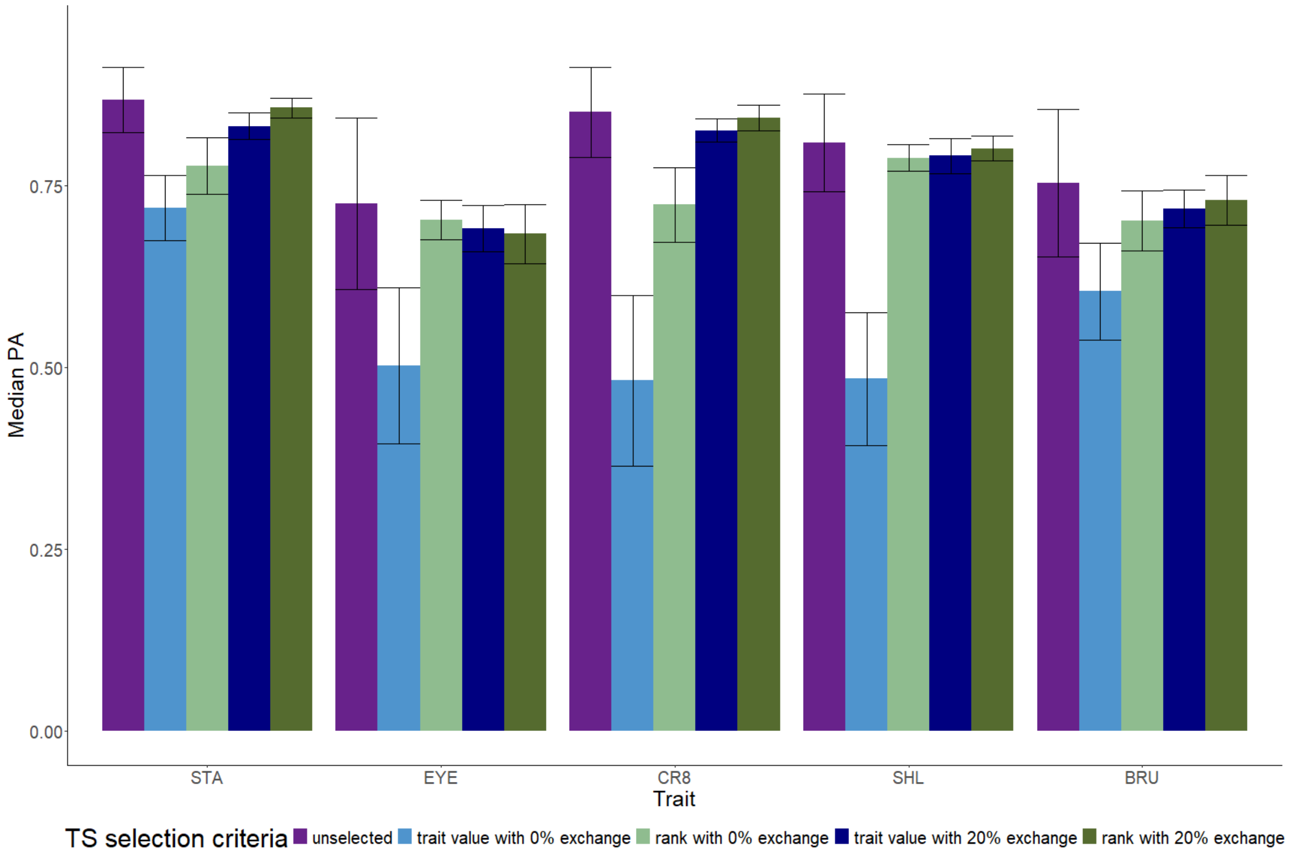

Fig. S11. Median prediction accuracy (PA) and the respective coefficient of variation (CoV) for five traits in a combination relevant for breeding when sampling the clones for the training set (TS) of the clones with 50% best trait values or ranks and additionally exchanging 0 or 20% of clones in the TS with clones that belong to the clones with 10% worst trait values or ranks. Here, rank defines the combined rank as the sum of single ranks for all traits, while the trait values were trait-specific. For abbreviations of the traits see Table 1.
